# A *Saccharomyces cerevisiae* Assay System to Investigate Ligand/AdipoR1 Interactions That Lead to Cellular Signaling

**DOI:** 10.1371/journal.pone.0065454

**Published:** 2013-06-07

**Authors:** Mustapha Aouida, Kangchang Kim, Abdul Rajjak Shaikh, Jose M. Pardo, Jörg Eppinger, Dae-Jin Yun, Ray A. Bressan, Meena L. Narasimhan

**Affiliations:** 1 Plant Stress Genomics Research Center, King Abdullah University of Science and Technology, Thuwal, Kingdom of Saudi Arabia; 2 Division of Applied Life Science (Brain Korea 21 Program), Plant Molecular Biology and Biotechnology Research Center, Gyeongsang National University, Jinju, Republic of Korea; 3 KAUST Catalysis Center, Division of Physical Sciences and Engineering, King Abdullah University of Science and Technology, Thuwal, Kingdom of Saudi Arabia; 4 Instituto de Recursos Naturales y Agrobiologia, Consejo Superior de Investigaciones Científicas, Sevilla, Spain; 5 Department of Horticulture and Landscape Architecture, Purdue University, West Lafayette, Indiana, United States of America; Queensland University of Technology, Australia

## Abstract

Adiponectin is a mammalian hormone that exerts anti-diabetic, anti-cancer and cardioprotective effects through interaction with its major ubiquitously expressed plasma membrane localized receptors, AdipoR1 and AdipoR2. Here, we report a *Saccharomyces cerevisiae* based method for investigating agonist-AdipoR interactions that is amenable for high-throughput scale-up and can be used to study both AdipoRs separately. Agonist-AdipoR1 interactions are detected using a split firefly luciferase assay based on reconstitution of firefly luciferase (Luc) activity due to juxtaposition of its N- and C-terminal fragments, NLuc and CLuc, by ligand induced interaction of the chimeric proteins CLuc-AdipoR1 and APPL1-NLuc (adaptor protein containing pleckstrin homology domain, phosphotyrosine binding domain and leucine zipper motif 1-NLuc) in a *S. cerevisiae* strain lacking the yeast homolog of AdipoRs (Izh2p). The assay monitors the earliest known step in the adiponectin-AdipoR anti-diabetic signaling cascade. We demonstrate that reconstituted Luc activity can be detected in colonies or cells using a CCD camera and quantified in cell suspensions using a microplate reader. AdipoR1-APPL1 interaction occurs in absence of ligand but can be stimulated specifically by agonists such as adiponectin and the tobacco protein osmotin that was shown to have AdipoR-dependent adiponectin-like biological activity in mammalian cells. To further validate this assay, we have modeled the three dimensional structures of receptor-ligand complexes of membrane-embedded AdipoR1 with cyclic peptides derived from osmotin or osmotin-like plant proteins. We demonstrate that the calculated AdipoR1-peptide binding energies correlate with the peptides’ ability to behave as AdipoR1 agonists in the split luciferase assay. Further, we demonstrate agonist-AdipoR dependent activation of protein kinase A (PKA) signaling and AMP activated protein kinase (AMPK) phosphorylation in *S. cerevisiae*, which are homologous to important mammalian adiponectin-AdipoR1 signaling pathways. This system should facilitate the development of therapeutic inventions targeting adiponectin and/or AdipoR physiology.

## Introduction

Adiponectin is a protein hormone secreted by adipose tissue in mammals and it plays crucial roles in the regulation of glucose and lipid metabolism, inflammation and oxidative stress [1], [2], [3], [4]. Missense mutations in the adiponectin gene are associated with the incidence of type 2 diabetes [5], [6]. Reduced serum concentrations of adiponectin correlate with obesity, type 2 diabetes, cardiovascular risk, as well as increased risk of several types of cancer [7], [8], [9]. Administration of recombinant adiponectin or genetic overexpression of adiponectin in rodent models has therapeutic effects in diabetes, ischemic cardiac injury and in colon cancer [10], [11], [12], [13], [14], [15], [16]. These findings triggered the great attention adiponectin receives today and support the development of therapeutic agents based on adiponectin replacement.

Adiponectin is a 30 kDa protein that consists of a N-terminal collagen domain and a C-terminal globular C1q domain [3]. It is a highly abundant serum protein. In serum, adiponectin is glycosylated and exists as a trimer, hexamer and higher order oligomers. The globular C1q domain can be released by proteolysis and is biologically active, but it is not found in significant amounts in serum. Several *in vitro* and *in vivo* studies have shown that two proteins, AdipoR1 and AdipoR2, serve as the major receptors for adiponectin [11], [17]. AdipoRs and G-protein coupled receptors (GPCRs) share a 7-transmembrane pass topology but are functionally distinct. Also, as members of the PAQR (Progestin and AdipoQ Receptor) family, AdipoRs have an cytosolic N-terminus and external C-terminus, which is the opposite orientation of classical GPCRs [18].

The signaling pathways activated by adiponectin depend on (a) the molecular form (glycosylation status and degree of oligomerization) of adiponectin, since the different forms show selectivity for the two AdipoRs, (b) the relative abundance of the two AdipoRs, since the receptors show selectivity for downstream signaling, and (c) the target tissue [17], [19], [20], [21]. For example, AdipoR1 acts as a high affinity receptor for globular adiponectin and has a lower affinity for full length adiponectin, while AdipoR2 possesses medium affinity for these adiponectin variants [21]. In skeletal muscle cells, the anti-diabetic effect of adiponectin is mediated by stimulation of glucose oxidation *via* the AMP activated protein kinase (AMPK) pathway and by increase of fatty acid oxidation *via* the AMPK and peroxisome proliferator-activated receptor α (PPARα) pathways [3], [20]. In the liver, the anti-diabetic action of adiponectin is linked to increased gluconeogenesis via AMPK activation, whereas stimulation of fatty acid oxidation, decrease of inflammation and increased insulin sensitivity are achieved *via* the PPARα pathway [17]. In vascular endothelial cells adiponectin has an anti-inflammatory effect due to enhanced nitric oxide production, suppression of oxidative stress and suppression of inflammatory signaling [22]. These anti-inflammatory effects of adiponectin in the vascular endothelium have been explained by AMPK activation and a cyclic AMP-protein kinase A (cAMP-PKA) pathway. In cardiomyocytes, the protective effects of adiponectin are mediated by AMPK and cyclo-oxygenase 2 signaling [22]. AdipoR1 exerts the major effect in mediating the anti-diabetic action of adiponectin in skeletal muscle whereas both AdipoRs have major roles in its anti-diabetic actions in the liver [17], [20].

These studies suggest that there is considerable value in the identification of AdipoR agonists as therapeutic agents. It is also evident that identification of signaling pathways specifically activated by an interaction between an agonist and one of the AdipoRs would provide valuable information for the design of therapeutic agents. We reasoned that a system designed to perform such studies in the budding yeast *Saccharomyces cerevisiae* would combine several advantages as detailed below.

**Figure 1:**
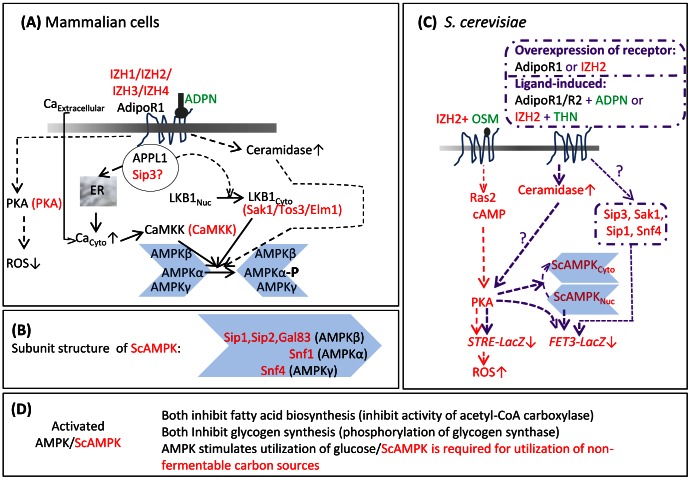
Summary of AdipoR-mediated signaling pathways in mammalian cells and IZH2-mediated signaling pathways in *S.* *cerevisiae* cells. (A) The components of the AdipoR1 signaling pathway of mammalian cells are shown in black font and their *S. cerevisiae* homologs, if known are shown in red font. In mammalian cells, adaptor protein containing pleckstrin homology domain, phosphotyrosine binding domain and leucine zipper motif 1 (APPL1) interacts directly with AdipoR1. Interaction of adiponectin (ADPN) with AdipoR1 stimulates AdipoR1-APPL1 interaction. This results in release of Ca^2+^ from the ER to the cytosol and also increases export of LKB1 kinase from the nucleus. Influx of extracellular Ca^2+^ to the cytosol is also stimulated by adiponectin, although the mechanism by which this occurs remains to be clarified. Increase in cytosolic Ca^2+^ concentration activates Ca^2+^/calmodulin-dependent protein kinase kinase (CaMKK) which in turn activates AMP activated protein kinase (AMPK) by phosphorylating its α subunit. However, phosphorylation of AMPK α subunit by the cytosol-localized kinase LKB1 is the major pathway for activation of AMPK. Adiponectin-AdipoR1 interaction also increases cellular ceramidase activity which in turn leads to phosphorylation of AMPKα subunit. The details of this pathway are not clear yet. Activation of AMPK is required for many of the anti-diabetic and anti-atherosclerotic effects of adiponectin. In vascular endothelial cells, interaction of AdipoR1 with adiponectin activates protein kinase A (PKA) which has the effect of lowering accumulation of reactive oxygen species (ROS) and thereby reducing inflammation. (B) Subunit structure of *S. cerevisiae* AMPK (ScAMPK). Like mammalian AMPK, ScAMPK is a trimer composed of an α, β, and γ subunit. The genes encoding the β subunit isoforms as well as the sole α and γ subunits are indicated. (C) Components of the *IZH2*-mediated signaling pathways in *S. cerevisiae* are shown in red font. Interaction of IZH2 (homolog of AdipoRs) with osmotin (OSM) activates PKA *via* a RAS2-cAMP pathway. Overexpression of *IZH2*, overexpression of *AdipoR1*, treatment of *S. cerevisiae* cells expressing *AdipoR1* with adiponectin and treatment of *S. cerevisiae* cells expressing various levels of *IZH2* with thaumatin (THN, a homolog of osmotin) has been shown to activate PKA by increasing cellular ceramidase activity. Activation of PKA leads to decreased transcription from a stress responsive promoter element (*STRE*) and increased cellular ROS content. Activated PKA promotes export of ScAMPK from the nucleus which leads to decreased transcription from the ferroxidase *(FET3*) promoter. Activated PKA also represses *FET3* transcription *via* the stress-responsive transcription factors MSN2/4. Mutational analyses show that genes encoding the APPL1-lke protein Sip3, the LKB1-like protein Sak1, the ScAMPK β subunit Sip1 and the ScAMPK γ subunit Snf4 are components of the pathway leading from *IZH2* (or *AdipoR1*) to *FET3* repression in *S. cerevisiae*.

First, reactions and regulatory steps of glucose and lipid metabolism are very similar in yeast and mammalian cells [23]. Energy metabolism in *S. cerevisiae* is connected to glucose and lipid metabolism *via* the same pathways that exist in mammalian cells. Importantly, AMPKs of mammals and *S. cerevisiae* have similar subunit structure [24], 25. *S. cerevisiae* and mammalian AMPKs can be cross-activated by their respective upstream kinases due to sequence similarity between the corresponding subunits. *S. cerevisiae* and mammalian AMPKs play similar roles in balancing energy demand with energy supply from carbohydrate and lipid metabolism (Figure 1).

Second, although four *S. cerevisiae* proteins Izh1p, Izh2p, Izh3p, and Izh4p share deduced sequence similarity with mammalian AdipoRs, adiponectin is unable to stimulate intracellular signaling that leads to repression of ferroxidase (*FET3*), a gene involved in metal ion homeostasis in yeast, unless AdipoR1 or AdipoR2 are expressed [26]. These results demonstrate that it is possible to examine signaling from AdipoR1 and AdipoR2 separately in yeast.

Third, activation of PKA and regulation of gene expression by AMPK due to adiponectin-AdipoR interaction contribute to the beneficial effects of adiponectin in mammalian systems [3], [22], [24]. Signaling from Izh2p and AdipoRs has also been shown to activate the PKA pathway and affect AMPK-dependent gene expression in *S. cerevisiae* (Figure 1). Izh2p and AdipoRs negatively regulate expression of genes involved in metal ion homeostasis through both PKA and AMPK in *S. cerevisiae*, *via* a CCCTC promoter element [26], [27]. It should be noted activation of PKA has an opposite effect on the cellular reactive oxygen species (ROS) content in mammalian and yeast systems. Activation of the AdipoR-PKA pathway decreases cellular content of ROS in mammalian vascular endothelial cells [22]. On the other hand, activation of the IZH2-PKA pathway signaling has been shown to suppress transcription from the CCCCT stress-responsive promoter element (STRE) that is found on some oxidative stress inducible genes, which increases the cellular content of ROS in *S. cerevisiae* [27], [28], [29].

However, adiponectin-AdipoR dependent activation of AMPK *via* increased phosphorylation of the *S. cerevisiae* AMPKα subunit, Snf1p, has not yet been described. Since this activating phosphorylation plays a key role in the anti-diabetic actions of adiponectin in skeletal muscle and liver, and in the cardioprotective actions of adiponectin [3], [19], [22], evidence for a central element in yeast adiponectin-AdipoR signaling is missing. Hence, we set out to establish *S. cerevisiae* systems to (a) accurately report agonist-AdipoR interactions and (b) report the agonist-AdipoR dependent activation of signaling pathways similar to those known to be important for controlling diabetes or cardiovascular function in mammals. Here we report a split firefly luciferase based assay for investigating ligand-AdipoR1 interactions. We further show that activation of yeast AMPK by phosphorylation of its α subunit, Snf1p, can be observed in response to agonist-AdipoR1 or agonist-AdipoR2 interaction in *S. cerevisiae* and demonstrate agonist-AdipoR dependent suppression of oxidative stress signaling *via* activation of the PKA pathway in *S. cerevisiae*. The split luciferase assays can be adapted easily for multiwell arrays suitable for high-throughput platforms using automated replica plating and mechanized washing. Hence our *S. cerevisiae* based system promises to be an effective tool for the identification of novel pharmaceutically active AdipoR agonists. It will also facilitate structure-function studies of AdipoRs and analyses of the influence of ligand structure on ligand-AdipoR interactions, which could serve as basis for intelligent agonist synthesis.

## Materials and Methods

### Strains and Growth Media

*S. cerevisiae* strains were derivatives of BWG1-7a *MATa ade1-100 ura3-52 leu2-3,112 his4-519* GAL+ [30] or BY4741 (*MATa his3*Δ*1 leu2*Δ*0 met5*Δ*0 ura3*Δ*0*) (from Dr. D. Ramotar, Universite de Montreal). Yeast cells were grown at 30°C in either YPD (1% yeast extract, 2% peptone, 2% dextrose) or selective synthetic complete medium [0.67% yeast nitrogen base without amino acids, 2% dextrose, pH 5.7, supplemented with appropriate dropout mix (Clontech, Mountain View, CA, USA)]. For control of gene expression under the *GAL* promoter, dextrose was substituted by raffinose and/or galactose to a final concentration of 2%. Yeast cells were transformed by the lithium acetate method [31]. Targeted insertion or deletion mutations of *IZH2*, *SNF1*, and *SIP3* were made using the *Kan*MX4 gene as described [32] and confirmed by PCR.

### Construction of Plasmids

Construction of the plasmid pSTRE-*lacZ* (*LEU2*) has been described earlier [29]. The entire coding regions of the human adiponectin receptors AdipoR1 and AdipoR2 and Adaptor protein containing Pleckstrin homology domain, Phosphotyrosine binding domain and Leucine zipper motif 1 (APPL1) were obtained by RT-PCR from total RNA isolated from MCF-7 breast cancer cells. The sequences of the primers used in the constructions described below are shown in Table S1. The PCR products obtained using primer pairs AdipoR1-F-Spe1/AdipoR1-R-Cla1 and AdipoR2-F-Spe1/AdipoR2-R-Cla1 were inserted into an intermediate vector by TA cloning, then excised with SpeI and ClaI and inserted into the corresponding sites of p426-GPD for construction of the plasmids p426-GPD-AdipoR1 and p426-GPD-AdipoR2. APPL1 coding sequence was amplified using primers APPL1-F-KpnI-SpeI and APPL1-R-SalI-1. The PCR product was cut with KpnI and SalI and inserted between the corresponding sites in vector 35S-NLuc [33], yielding the chimeric 35S-APPL1-NLuc construct. The APPL1-NLuc fragment was then excised by digestion with SpeI and PstI and ligated into p425-GPD cut with the same enzymes to generate the p425-GPD-APPL1-NLuc construct. Construction of pESC-CLuc-AdipoR1 and pESC-CLuc-AdipoR2 was also performed in two steps. CLuc was first introduced downstream of the *GAL1* promoter in pESC-URA and then AdipoR1 or AdipoR2 were fused in-frame to CLuc. CLuc was amplified by PCR from the vector 35S-CLuc using primers CLuc-F-BamHI and CLuc-R-XhoI-AscI-HindIII. The PCR products were digested with BamHI and HindIII and cloned into the cognate sites of pESC-URA, yielding pESC-CLuc. Then the coding sequence of AdipoR1 and AdipoR2 were amplified by RT-PCR from the total RNA using the primer pairs AdipoR1-F-XhoI/AdipoR1-HindIII and AdipoR2-F-XhoI/AdipoR2-HindIII, respectively. The PCR products were digested with XhoI and HindIII and ligated into pESC-CLuc digested with the same enzymes to yield pESC-CLuc-AdipoR1 and pESC-CLuc-AdipoR2. pESC-CLuc-AdipoR1-APPL1-NLuc that contains *GAL1-*CLuc-AdipoR1 and *GAL10*-APPL1-NLuc in the same vector was constructed as follows. APPL1-NLuc was amplified from p425-GPD-APPL1-NLuc by PCR using the primer pair APPL1-F-NotI/NLuc -R-ClaI. The PCR product was digested with NotI and ClaI inserted into the cognate sites pESC-CLuc-AdipoR1 yielding pESC-CLuc-AdipoR1-APPL1-NLuc. All constructs were verified by sequencing.

The pYES-EGFP-AdipoR1 and pYES-EGFP-AdipoR2 plasmids that contained EGFP fused in-frame to the N-terminus of AdipoR1 and AdipoR2, respectively, were constructed as follows. The entire AdipoR1 and AdipoR2 ORFs were amplified by PCR from cDNA isolated from human THP-1 cells using the AdipoR1-F-GFP/AdipoR1-R-GFP and AdipoR2-F-GFP/AdipoR2-R-GFP primer pairs shown in Table S1. pYES-EGFP vector (digested with BamHI and XhoI) mixed with the appropriate the PCR product (digested with EcoR1 and Xho1) was transformed into *S. cerevisiae* strain BY4741 for gap repair cloning. Positive clones were confirmed by sequencing.

### Osmotin, Adiponectin and Peptides

Osmotin was purified to homogeneity from suspension cultured salt-adapted tobacco cells as described before [34]. Osmotin was stored as sterile solution in ultrapure water (HPLC grade). The osmotin preparation was determined to be over 99% homogenous by 2D polyacrylamide gel electrophoresis and by mass spectrometry. The IC_50_ (amount of osmotin that reduces growth by 50%) was determined by literature methods [35] and ranged between 4–8 µg/mL for the osmotin batches used in this study. Osmotin and adiponectin were quantified using the BCA Protein Assay Reagent (Pierce, Rockford, IL, USA) using bovine serum albumin as standard. Unless specified otherwise, the pure recombinant *Escherichia coli* expressed N-terminal (His)_6_-tagged full length human adiponectin used in these studies was the gift of Dr. A. Sharkhuu (KAUST, Saudi Arabia) or Dr. I. Sokolchik (Purdue University, USA). The biological activity of endotoxin-free full length human adiponectin batches used in this work was verified by measuring its ability to induce apoptosis of MCF-7 breast cancer cells or THP-1 monocytic leukemia cells [36], [37]. Bacterially expressed human globular adiponectin (Biovendor, Candler, NC, USA) and bacterially expressed recombinant full length human adiponectin (Biovision, Mountain View, CA, USA or Genway, San Diego, CA, USA) were used in some tests as indicated in the figures. The synthetic cyclic peptides, OSMpep and ZMTNpep, used in this worked were purchased from American Peptide Company (Sunnyvale, CA, USA). Peptide purity was 98% and they were dissolved in water at concentration of 1 mg/mL.

### Gene Expression Analysis by RT-PCR

Total RNA was prepared using the RiboPure-Yeast extraction kit (Ambion, Grand Island, NY, USA) from 3 mL of overnight culture grown in selective minimal medium and genomic DNA contamination was eliminated using the TURBO DNA-free kit (Ambion). cDNA was synthesized from total RNA (2 µg) using M-MLV reverse transcriptase (Invitrogen, Grand Island, NY, USA). The PCR primers used to amplify the target genes are listed in Table S2. The PCR program was 2 min at 95°C followed by 25 cycles of 1 min of denaturation at 94°C, 2 min of annealing at a temperature specific for each gene fragment (*e.g.* 54°C for *ACT1* fragment) and 4 min of extension at 72°C. This was followed by extension at 72°C for 7 min. PCR products were analyzed on a 1% agarose gel.

### Luminescence Imaging and Luciferase Activity Measurement

Cultures were grown overnight at 30°C in selective minimal with 2% raffinose as carbon source. Cells were collected by 5 min centrifugation at 6000 rpm, and cell pellets were washed with the same medium but lacking sugar. Cells were resuspended at A_600 nm_ of 0.4 in selective minimal medium supplemented with galactose or a galactose-raffinose mixture (2% sugar) as carbon source and grown at 30°C for 16 h. The cultures were adjusted to A_600 nm_ of 0.4 and aliquots (100 µL) were centrifuged for 1 min at 16,000×g. Cell pellets were resuspended in 100 µL of 0.1 M Na citrate buffer pH 3.0 containing 1 mM D-luciferin (Gold Biotechnology, St. Louis, MO, USA), and transferred to individual wells of a 96-well plate (Nunc White polystyrene). The assay plate was incubated for 5 min at room temperature in the darkness before luminescence imaging under a CCD camera (Lumazone, Princeton Instruments, USA) or for luciferase activity measurement using a TECAN Ultra 96microplate reader. A unit of luciferase activity was calculated as the luminescence value of sample minus the luminescence value of blank solvent.

### Measurement of β-Galactosidase Activity

For determination of β-galactosidase activity, cells were grown in selective minimal medium to A_600 nm_ of 0.4, resuspended in YPD at A_600 nm_ of 6.0 (ca. 10^8^ cells/mL). Cells (1 mL) were then mixed with water (0.2 mL) containing various amounts of osmotin and incubated at 30°C with occasional mixing. Cells were collected at the end of the incubation period by centrifugation and resuspended in the same volume of ice cold Z buffer (60 mM Na_2_HPO_4_, 40 mM NaH_2_PO_4_, 10 mM KCl, 1 mM MgSO_4_, 50 mM 2-mercaptoethanol, pH 7.0). The A_600 nm_ of the cell suspension was measured and adjusted to 1.0 by the addition of more ice cold Z buffer. Cells (0.1 mL) were mixed with Z buffer (0.9 mL), chloroform (0.1 mL) and 0.1% SDS (5 µL), vortexed for 10 sec and then subjected to three freeze-thaw cycles (liquid nitrogen 30 sec–37°C 90 sec). The suspension of broken cells was warmed to 28°C and enzyme activity was initiated by addition of *o*-nitrophenyl-β-D-galactopyranoside (0.2 mL of 4 mg/mL solution). The reaction was allowed to proceed at 28°C for various time periods and then terminated by addition of 1 M sodium carbonate solution (0.5 mL). The suspension was clarified by centrifugation and the absorbance of the supernatant was measured at 420 nm. One unit of β-galactosidase activity was defined as 1000 X (ΔA_420 nm_/min/A_600 nm_).

### Immunoblot Analysis

For examining the expression of EGFP-tagged AdipoRs, cultures were grown as described under ‘Luminescence Imaging and Luciferase Activity Measurement’. Total protein extracts were prepared from cells grown on 2% galactose in yeast extraction buffer (50 mM Tris-HCl pH 8.0, 50 mM NaCl, 5% glycerol, 1 mM each of EDTA, phenylmethylsulfonyl fluoride, and dithiotheritol, 0.5 µg/mL each of pepstatin, aprotinin, and leupeptin) as previously described [38]. One hundred µg of membrane proteins were fractionated by 10% SDS-PAGE and transferred to a polyvinylidene difluoride (PVDF) membrane (Immobilon P; Millipore). EGFP was visualized by the ECL method (GE Healthcare, UK) using monoclonal antibody to GFP as primary antibody (Clontech, Mountain View, CA, USA) and horseradish peroxidase-conjugated secondary antibody.

For examining the phosphorylation of *S. cerevisiae* AMPKα subunit (Snf1p), cells were grown and treated with osmotin (or adiponectin) exactly as described under ‘Measurement of β-Galactosidase Activity’. Treated cells were broken by trichloroacetic acid (TCA) protein extraction method and cell-free extracts were fractionated by SDS-PAGE on 10% polyacrylamide gels. After transfer to nitrocellulose membranes (Millipore, Billerica, MA, USA), phospho-Snf1p was detected by the ECL method (Thermo, Rockford, IL, USA) using monoclonal antibody to phospho-AMPKα (Thr172) as the primary antibody (Cell Signaling, Danvers, MA, USA) and goat anti-rabbit horseradish peroxidase as secondary antibody. Band intensities were measured on Luminescent Image Analyzer LAS-4000 with Multi Gauge V3.0 program (Fuji film, Tokyo, Japan). For determination of osmotin sensitivity, cells were washed three times with YPD at the end of the incubation period and serial dilutions in water were spotted on YPD agar plates.

### Confocal Microscopy

Cells expressing EGFP-tagged receptors were grown as described under ‘Luminescence Imaging and Luciferase Activity Measurement’. Cells grown in 2% galactose were fixed by incubating in 0.1% formaldehyde at room temperature for 25 min. After washing 3 X with 0.1 M sodium phosphate buffer containing 0.137 M NaCl (pH 6.5), cells were re-suspended in the same buffer and stained for 5 minutes with DAPI (0.5 µg/mL) in the dark at room temperature. Cells were examined and photographed under a confocal laser-scanning microscope (Zeiss LSM 510 META).

### Computational Studies

Details on homology modeling, MD simulations and docking studies are given in Methods S1. In brief, the 3D-homology model of human AdipoR1 was built for amino acids 135–364 with the Schrödinger suite 2011 using archeal (*Natronomonos pharaonis*) phototaxis protein rhodopsin II (PDB id: 1GU8) as template structure [39]. The resulting homology model of AdipoR1 was embedded in 1,2-dipalmitoylphosphatidylcholine (DPPC) membrane and subjected to minimization and MD relaxation [GROMACS 4.5.5 software package and GROMOS53a6 force field [40]. The 3D structure of the globular domain of human adiponectin (residues 105–254) was built using the YASARA structure molecular modeling package [41] using a murine isoform (PDB id: 1C3H) as template [42]. Energy minimization and MD relaxation was performed using Yasara scripts. For docking of protein and peptide/AdipoR1 complexes PATCHDOCK and FireDock were used. Resulting complexes were energy minimized and analyzed following MD simulation (GROMACS).

## Results

### Adiponectin Receptors are Expressed on the Surface of Yeast Cells

**Figure 2:**
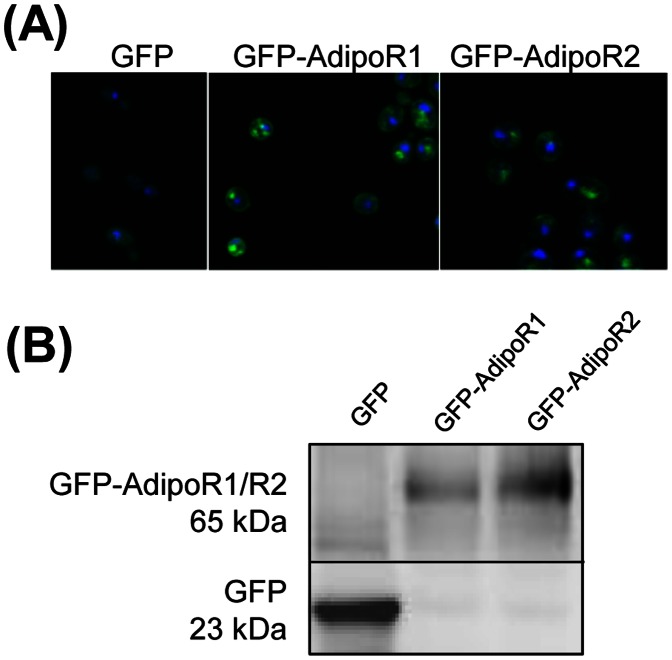
AdipoRs are expressed on the plasma membrane in *S. cerevisiae*. (A) Subcellular localization of AdipoRs by confocal microscopy. *S. cerevisiae* strain BY4741 carrying plasmid pYES-EGFP (GFP), pYES-EGFP-AdipoR1 (GFP-AdipoR1) and pYES-EGFP-AdipoR2 (GFP-AdipoR2) were cultured in selective minimal medium containing 2% galactose. Shown are images of cells that were in the early log phase of growth. (B) Western blot analysis of total membrane protein extracts that were fractionated by 10% SDS-PAGE. The predicted molecular weights of GFP-AdipoR1 and GFP-AdipoR2 are around 65 kDa.

We first confirmed the correct localization of AdipoRs in plasma membranes of *S. cerevisiae* by fluorescence microscopy using EGFP-AdipoR fusion proteins. As shown in Figure 2, cells of the wild type *S. cerevisiae* strain BY4741 transformed with a multicopy plasmid expressing EGFP under the *GAL* promoter displayed a diffuse intracellular fluorescence suggestive of cytoplasmic localization. However, cells expressing EGFP-AdipoR1 or EGFP-AdipoR2 from the same plasmid vector exhibited a strong fluorescence on the surface that was absent in control cells expressing only EGFP, suggesting plasma membrane localization of the recombinant AdipoRs. Fluorescence was also observed in vesicles that resembled the pre-vacuolar compartment, possibly due to degradation of AdipoR1 and AdipoR2 in the vacuole. Expression of EGFP-AdipoR1 (65 kDa), EGFP-AdipoR2 (65 kDa) and EGFP (26 kDa) was confirmed by Western blot analysis of cell-free extracts with an anti-GFP antibody.

### Strategy for Reporting Adiponectin-AdipoR1 Interaction

**Figure 3:**
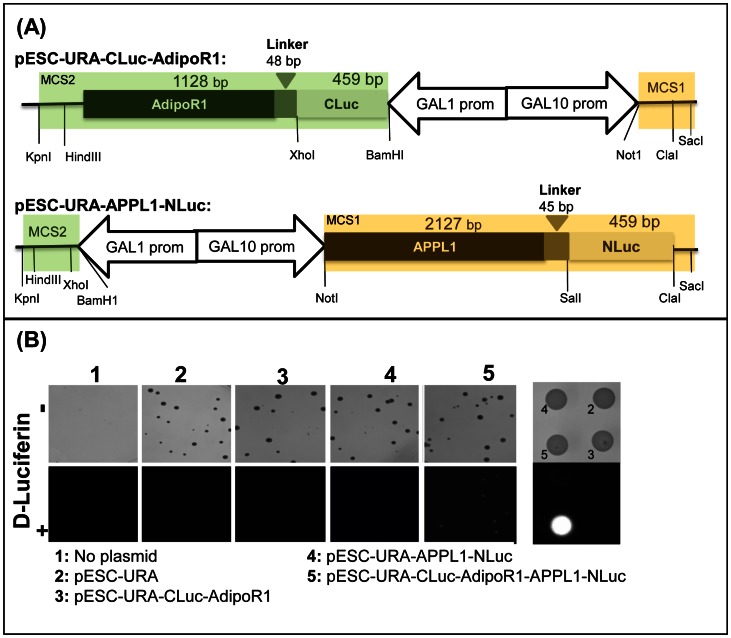
The split firefly luciferase complementation colony assay for AdipoR1 and APPL1 interaction. (A) Schematic representation of the plasmid vector constructs. CLuc-AdipoR1 was cloned into the multiple cloning site 2 (MCS2, green shadow) of pESC-URA for expression from the *GAL1* promoter and Appl1-NLuc was cloned into MCS1 (orange shadow) of pESC-URA for expression from the *GAL10* promoter. pESC-URA-CLuc-AdipoR1-APPL1-NLuc (not shown) contains the depicted APPL1-NLuc construct inserted between Not1 and Sac1 sites of the MCS1 of pESC-URA-CLuc-AdipoR1. (B) Visualization of the AdipoR1-APPL1 interaction in colonies. Cells of strain BY4741 untransformed (No plasmid) or transformed with the indicated plasmids were spread on minimal medium lacking uracil containing 2% galactose. After 2 days incubation at 30°C, plates were photographed before (–) and imaged after (+) spraying with D-luciferin.

In mammalian cells, APPL1 directly interacts with the intracellular N-terminal sequence of AdipoR1 [43], [44]. AdipoR1-APPL1 interaction is stimulated by adiponectin and it triggers the activation of AMPK in mammalian cells (Figure 1). APPL1 also interacts with AdipoR2 [43], but downstream signaling resulting from the AdipoR2-APPL1 interaction is not well-understood. Since ligand-stimulated AdipoR1-APPL1 interaction is the earliest known step in the adiponectin-AdipoR signaling cascade, we decided to use it as the basis for our *S. cerevisiae-*based assay targeting AdipoR1-ligand interactions. We selected the split-luciferase bimolecular complementation assay as reporter system for AdipoR1-APPL1 contacts, since it is a well-established method for studying protein-protein interactions in mammalian, plant and *S. cerevisiae* cells [45], [46], [47]. Accordingly, a chimeric gene encoding human APPL1 fused in-frame at its C-terminus to firefly luciferase fragment 2-416 (NLuc) was cloned into the multiple cloning site 1 of the vector pESC-URA to yield the control plasmid pESC-URA-APPL1-NLuc (Figure 3A). This plasmid allows the expression of APPL1-NLuc from the *GAL10* promoter. Similarly, a second chimeric gene encoding firefly luciferase fragment 398–550 (CLuc) fused to the N-terminus of AdipoR1, and inserted into the multiple cloning site 2 of the vector pESC-URA, yielded the control plasmid pESC-URA-CLuc-AdipoR1 (Figure 3A). This plasmid allows the expression of CLuc-AdipoR1 from the *GAL1* promoter. Both chimeric proteins, CLuc-AdipoR1 and APPL1-NLuc, contained a linker between the two fragments to confer some mobility to the luciferase domains thus facilitating reconstitution of luciferase activity. The test plasmid pESC-URA-CLuc-AdipoR1-APPL1-NLuc for simultaneous expression of both the chimeric proteins, APPL1-NLuc and CLuc-AdipoR1, was then constructed from these two control plasmids using gap repair cloning. All plasmids were transformed into *S. cerevisiae* strain BY4741 for expression test.

### Proof-Of-Concept: Receptor Level-Dependent Reconstitution of Luciferase Activity

**Figure 4:**
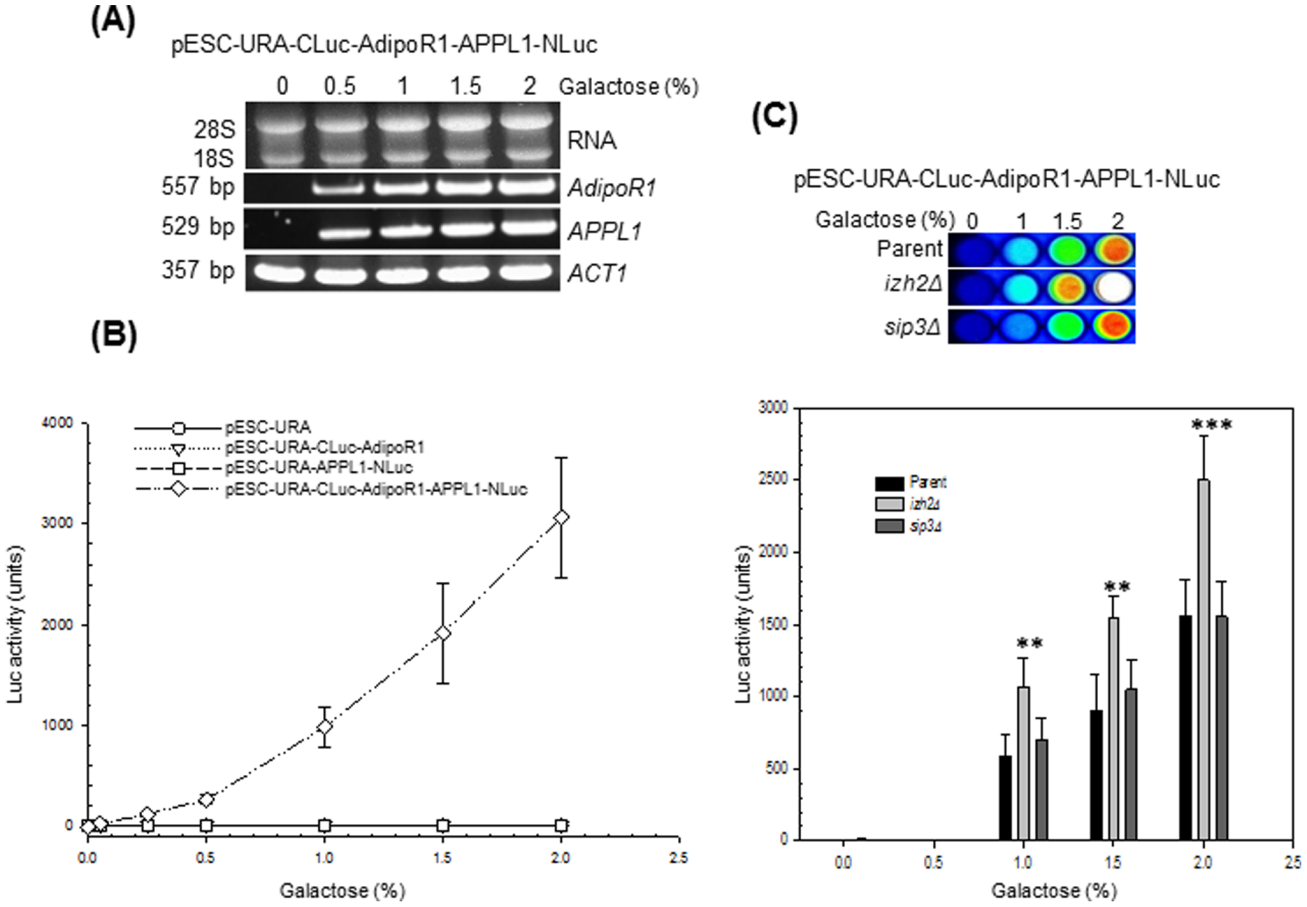
Luciferase reporter activity depends on the expression level of AdipoR1 and APPL1. (A) RT-PCR analysis of *AdipoR1* and *APPL1* expression in total RNA (2 µg) from cells of strain BY4741 carrying pESC-URA-CLuc-AdipoR1-APPL1-NLuc that were grown for 16 h at 30°C in selective minimal medium at the indicated galactose concentrations. The final concentration of sugars in the growth media was adjusted to 2% with raffinose. *ACT1* expression is shown for normalization. (B) Quantification of luciferase activity in BY4741 cells carrying pESC-URA, pESC-URA-CLuc-AdipoR1, pESC-URA-APPL1-NLuc and pESC-URA-CLuc-AdipoR1-APPL1-NLuc plasmids. Cells were grown for 16 h at 30°C in selective minimal medium at the indicated galactose concentrations. Luciferase activity measurements were made as described in Methods. (C) Imaging (top) and quantification of luciferase activity (bottom) in pESC-URA-CLuc-AdipoR1-APPL1-NLuc transformants of strain BY4741 (Parent) or its isogenic *izh2Δ* and *sip3Δ* derivative strains. Assays were performed as described above on cells that were grown for 16 h at 30°C in selective minimal medium at the indicated galactose concentrations. Results are expressed as the mean ± SD from three separate experiments with triplicate samples. Significant difference from the parent strain is indicated by asterisks. Symbols: **, *p*<0.01; ***, *p*<0.001.

It has been reported that overexpression of *IZH2* and *AdipoR1* can activate downstream signaling leading to suppression of *FET3* promoter-dependent transcription even in absence of a ligand (Figure 1) [26], [48]. Therefore, to prove that the bi-molecular complementation assay based on split-luciferase was functional, we performed luminescence imaging of colonies after full induction of the *GAL1* and *GAL10* promoters by 2% galactose in the growth media. It can be seen (Figure 3B) that upon application of the substrate, D-luciferin, only transformants carrying the test plasmid pESC-URA-CLuc-AdipoR1-APPL1-NLuc exhibited luminescence whereas transformants carrying either the vector pESC-URA, or the control plasmids pESC-URA-CLuc-AdipoR1 and pESC-URA-APPL1-NLuc did not. Next, we studied whether the obtained reporter gene activity is proportional to the expression levels of *AdipoR1* and *APPL1*. First, expression levels of both *AdipoR1* and *APPL1* in the pESC-URA-CLuc-AdipoR1-APPL1-NLuc transformant were confirmed to increase in proportion to the galactose concentration in the growth medium (Figure 4A). Next, assays performed in microtiter plates showed that luciferase activity in pESC-URA-CLuc-AdipoR1-APPL1-NLuc transformants increased as a function of galactose concentration in the medium (Figure 4B). Again, no significant luciferase activity could be detected in controls that expressed only one or none of the interaction partners (Figure 4B). These results indicated that the reporter activity directly correlated with the amounts of the interacting partners.

Among the IZH family proteins, Izh2p has most homology with AdipoRs (about 32% amino acid sequence identity). Therefore, we examined whether *IZH2* maybe interfering with the AdipoR1-APPL1 interaction resulting in a reduced luciferase reporter activity. As shown in Figure 4C, luciferase activity was increased significantly by inactivation of *IZH2*. This effect was observed in cells grown in 1%, 1.5% and 2% galactose, indicating that Izh2p competes with AdipoR1 for binding to APPL1. The putative ortholog of APPL1 in *S. cerevisiae* is Sip3p and genetic evidence supports a role of *SIP3* in *IZH2* signaling (Figure 1) [26]. However, inactivation of *SIP3* did not significantly increase luciferase activity indicating that Sip3p does not compete with APPL1 for AdipoR1 binding (Figure 4C).

### Demonstration of Adiponectin- and Osmotin-Induced Increase in Luciferase Activity

Next, we tested whether incubation of galactose-induced cells with AdipoR1 ligands further stimulated luciferase reporter activity. In addition to adiponectin, we also used osmotin as a test ligand. Osmotin is a tobacco defense protein that has been shown to be a structural and functional homolog of adiponectin [29]. Specifically, osmotin stimulates AMPKα phosphorylation in an AdipoR-dependent manner in murine skeletal muscle cells and also stimulates *IZH2*-dependent intracellular signaling in *S. cerevisiae* [29]. While direct binding of adiponectin and osmotin to AdipoRs has not yet been observed experimentally, homology model based docking results confirm that complexes of both proteins with the extracellular surface of AdipoR1 are energetically favorable [49].

**Figure 5:**
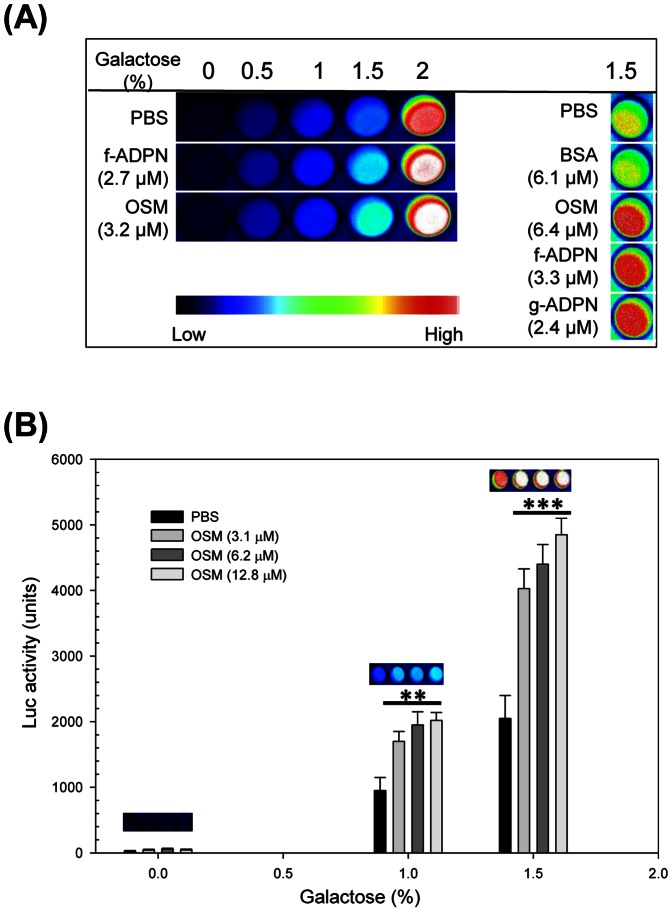
The AdipoR1 ligands, adiponectin and osmotin, induce increase in Luc reporter activity. Cells of strain BY4741carrying pESC-URA-CLuc-AdipoR1-APPL1-NLuc were grown for 16 h at 30°C in selective minimal medium at the indicated galactose concentrations, treated for 4 h at 30°C with the indicated test compounds and then assayed for Luc activity. (A) Imaging of Luc activity. (B) Quantitative measurement of Luc activity as a function of osmotin concentration. A representative image of relative Luc activity at the different osmotin concentrations is shown for each galactose concentration. Data represent the means ± SD from three experiments with triplicate samples. For each galactose concentration, significant differences by a Student’s t-test between osmotin treated samples and untreated control are indicated by asterisks. Symbols: PBS, 1/8 X PBS; f-ADPN, bacterially expressed full length adiponectin; BSA, bovine serum albumin, OSM, osmotin; g-ADPN, bacterially expressed globular adiponectin; **, *p*<0.01; ***, *p*<0.001.

Luciferase activity of galactose-induced pESC-URA-CLuc-AdipoR1-APPL1-NLuc carrying cells of strain BY4741 was increased upon additional incubation with either globular adiponectin, full length adiponectin from two sources or osmotin, compared to incubation with solvent (1/8 X PBS) (Figures 5A and S1). On the other hand, the luciferase activity remained constant upon additional incubation with the negative control protein Bovine Serum Albumin (BSA) compared to incubation with solvent (Figure 5A), indicating that the luciferase activity was stimulated specifically by the predicted ligands of AdipoR1 [49]. These results also agree with experiments showing that AdipoR1-signaling is initiated by bacterially expressed full length adiponectin, globular adiponectin and osmotin [21], [29]. Ligand-induced increase in luciferase activity was observed only upon achieving a detectable signal even in absence of added ligand, requiring cells to be induced with 1–2% galactose (Figures 5A and S1). Next, we quantified luciferase activity in cells induced with different galactose concentrations after incubation with a range of osmotin concentrations (Figure 5B). The results confirmed that ligand-induced luciferase activity was observed only in cells that were grown at or above 1% galactose. Luciferase activity was approximately the same at 1% and 1.5% galactose and also did not vary within the range of osmotin concentrations tested (3.2–12.8 µM).

### Evaluation of Thaumatin Like Protein (TLP)-Based Peptides as Ligands of AdipoR1

A nine residue cyclic osmotin-derived peptide (OSMpep, Table 1) was predicted to interact with AdipoR1 on the basis of molecular modeling studies and then shown to induce AdipoR1-dependent intracellular signaling in mammalian cells [49]. This peptide is structurally stable and rigid and the simulation of its interaction with AdipoR1 predicted that it binds to the same region of AdipoR1 as adiponectin [49]. Proteins with sequence, structure and serological similarity to osmotin are ubiquitous in plants and are referred to as thaumatin-like proteins (TLPs). We hypothesized that the homologs of OSMpep from other TLPs such as soybean P21, banana TLP, thaumatin, kiwi Act d2 and corn zeamatin may bind as well or better to AdipoR1 as OSMpep. These TLPs were selected as the source of peptide sequences for the following reasons: *(i)* the structures of osmotin, banana TLP, thaumatin and zeamatin have been determined by X-ray crystallography (Table 1), so many of the selected peptides are confirmed to exist in a similar structural context in all the TLPs; *(ii)* thaumatin and osmotin have been shown to stimulate *IZH2*-dependent signaling in *S. cerevisiae* and many TLPs have antifungal activity, rendering it possible that some of these peptides might have biological activity [29], [48], [50], [51]. We tested our hypothesis using a molecular modeling approach. Building on the strategy of Miele *et al*., [49], we were able to create a membrane embedded AdipoR1 homology model covering amino acids 135–364 of human AdipoR1. The 11 additional C-terminal amino acids in our model compared to that of Miele *et al*., [49] provided an extended 7^th^ transmembrane helix which reached up to the receptor’s binding site. This homology model served as basis for docking studies of adiponectin and osmotin on AdipoR1 and to predict TLP peptide-AdipoR1 interactions and possible TLP binding (Figures S2 and S3). Shortly after we had finished our molecular modeling studies, a crystal structure of an engineered single-chain trimer human adiponectin globular domain was published (PBD id: 4DOU) [58]. The structure is in excellent agreement with our homology model for human globular adiponectin with a nearly identical geometry of the binding site residues (Figure S3G). Our homology model and engineered trimeric templates [52]lead to nearly identical docking results.

**Table 1:** Docking score for peptides docked in AdipoR1.

Ligand	Protein/source of purified protein	PDB/UniProt	Peptide sequence^g^	score	E_bind_ (kcal/mol)
**OSMpep**	Osmotin^a^/Salt adapted tobacco cells	1PCV/P14170	CTQGPCGPT	−8.21	−94.89
**P21pep**	P21^b^/ Soybean leaf		CNSGSCGPT	−7.25	−78.21
**BANpep**	Ban-TLP^c^/banana fruit/	1Z3Q/O22322	CNSGSCSPT	−6.45	−76.88
**THNpep**	Thaumatin^d^/*Thaumatococcus danielli* (katemfe) fruit	1THV/P02883	CTTGKCGPT	−6.82	−88.72
**ACTd2pep**	Act d2^e^/Kiwi fruit/	−/P81370	CNSGNCGLT	−5.91	−76.49
**ZMTNpep**	Zeamatin^f^/corn kernel/	1DU5/P33679	CVGSAANDCHPT	−5.95	−71.48

a [56];

b [57];

c [58];

d [59];

e [60];

f [61];

g disulfide-bonded cyclic peptides.

All ligand/AdipoR1 interactions predicted by our model took place at the same overall binding site, which was formed by a central cleft between the 3 extracellular loops and extended to the C-terminal 7^th^ transmembrane helix of AdipoR1. Our membrane embedded model gave clear indications, identifying AdipoR1 residues that can participate in binding. For example, one top scoring complex for the docking of osmotin to the membrane free receptor model [49] was omitted in our membrane embedded model, because part of the interaction took place on the membrane-covered surface of AdipoR1. Although our membrane embedded model and the membrane free receptor model [49] predict similar overall architectures of complexes and binding sites, we found clear differences in the residues involved in ligand/AdipoR1 interactions (see Figures S4 and S5 for details). Compared to the earlier model [49], the interacting region of AdipoR1 in our model was generally shorter on loop 1, shifted to toward the C-terminus on loop 3, and extended on loop 5. We also found a weak C-terminal interaction. However amino acids G354 and V355 of AdipoR1, which are predicted to be interacting residues in the model of Miele et *al*., [49] were found to be membrane embedded and hence not accessible in our model. Overall, extension of helix 7 and membrane embedding led to important differences in the prediction of ligand binding sites on AdipoR1.

**Figure 6:**
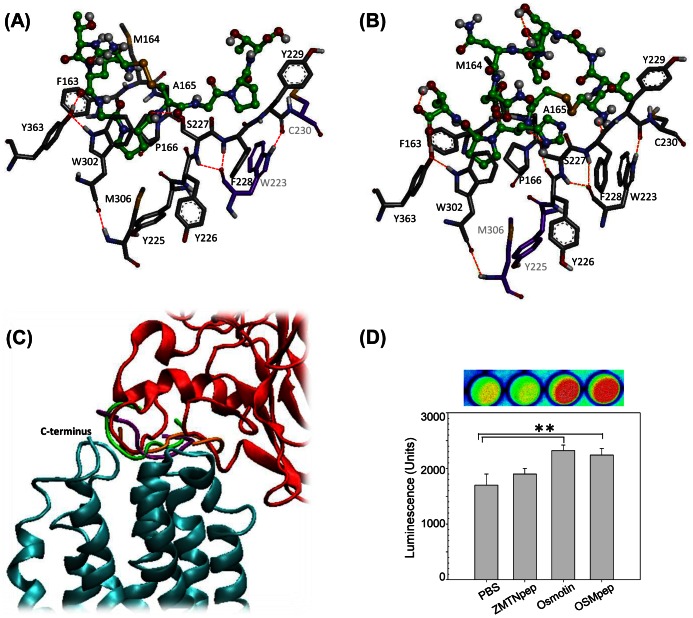
Comparison of TLP-derived peptide ligands binding to AdipoR1. 3D model of (A) OSMpep and (B) ZMTNpep bound to AdipoR1. Peptides are shown in ball and stick representation (C atoms in green). The interacting residues of AdipoR1 are shown as sticks only (interacting residues: C in gray; non-interacting residues: C in purple). Atom color coding is white = H, red = O, blue = N, yellow = S. (C) Overlay of osmotin (red) and the three top scoring OSMpep poses (peptide strands in green, purple and orange illustrate orientation of poses with 1^st^, 2^nd^ and 3^rd^ highest docking score) bound to AdipoR1 (blue). (D) Verification of interaction strength of the peptides with AdipoR1 by the split luciferase assay. Cells of strain BY4741carrying pESC-URA-CLuc-AdipoR1-APPL1-NLuc were grown for 16 h at 30°C in selective minimal medium containing 1.5% galactose, treated for 4 h at 30°C with the indicated test compounds and then used for luciferase activity measurement. The concentrations of test compounds in the assay were: PBS, 1/8 X PBS; ZMTNpep, 80 µg/mL; osmotin, 80 µg/mL (3.1 µM) and OSMpep, 80 µg/mL. Shown is a representative image of luciferase activity and quantification of the luminescence under the CCD camera. Data are the mean ±SD from three separate experiments with triplicate samples. Asterisks represent significant differences at **, *p*<0.01 by Student’s t-test.

The docking scores received for the tested TLP peptides suggest that the osmotin derived peptide (OSMpep) has the highest binding affinity to AdipoR1, while the zeamatin derived peptide (ZMTNpep) is the weakest binding ligand (Table 1). However, OSMpep and ZMTNpep interact in a very similar way with the AdipoR1 binding site, which also strongly resembles the AdipoR1-osmotin interaction (Figures 6, S3D, S4 and S6). We tested the model prediction experimentally using the split luciferase assay described above. The assay was performed in the *izh2Δ* mutant to avoid possible interference by Izhp. As shown in Figure 6D, the luminescence intensity of pESC-URA-CLuc-AdipoR1-APPL1-NLuc transformed cells treated with osmotin or OSMpep was significantly greater than that of cells treated with PBS or equivalent concentration of ZMTNpep. Thus, computational predictions and experimental results are in agreement, which suggests that the split luciferase assay is able to discriminate between good and poor ligands of AdipoR1.

### Osmotin Signaling Mediated by Adiponectin Receptors Results in Phosphorylation of Snf1p

**Figure 7:**
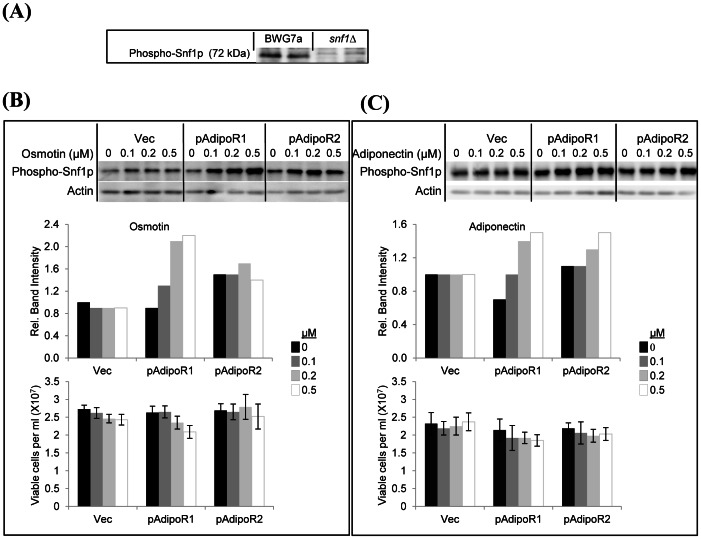
Osmotin signaling mediated by adiponectin receptors induces Snf1p phosphorylation in an *izh2Δ* mutant. (A) Cell lysates (100 µg protein) of strain BWG1-7a and an isogenic *Δsnf1::Kan_MX* line were fractionated by 10% SDS-PAGE. Shown are blots probed with anti-phospho-AMPK(Thr-172) antibody. The expected size of Snf1p is 72 kDa. (B, C) Cells (about 10^8^/mL) of strain BWG1-7a *Δizh2::Kan_MX* transformed with p426GPD (Vec), p426GPD-*AdipoR1* (pAdipoR1) or p426GPD-*AdipoR2* (pAdipoR2) were treated with indicated osmotin and adiponectin concentrations for 30 min at 30°C in YPD. Aliquots were withdrawn for viable counts determination before the cell lysates were prepared for analysis by10% SDS-PAGE. Shown in the top panels are blots probed first with phospho-AMPK(Thr-172) antibody, then stripped and probed with actin antibody (100 µg total protein per lane). Shown in the middle panels are relative band intensities in the depicted gels. ‘Relative band intensity’ was defined as the ratio of the intensity of phospho-Snf1p signal to actin signal for each lane when the value of this ratio for the corresponding untreated Vec sample was arbitrarily assigned the value 1.0. Shown in the bottom panels are viable counts in each sample at the end of the treatments. The experiments were performed twice with comparable results and the results of one experiment are shown.

AMPKs have conserved structure and are essential for control of energy metabolism in yeast, plants and mammals [24], [25]. The catalytic activity of AMPKs is upregulated by phosphorylation of a conserved Thr residue on the catalytic α subunit. Phosphorylation of murine AMPKα subunit on this conserved Thr^172^ by adiponectin or osmotin is abrogated by silencing the expression of adiponectin receptors in cultured murine myotubes [29]. Snf1p is the sole AMPKα subunit present in the *S. cerevisiae* proteome and it is activated by phosphorylation at the conserved threonine, Thr^210^, in response to glucose starvation or stress [25]. To examine whether osmotin and adiponectin can stimulate AdipoR1- or AdipoR2-dependent phosphorylation of Snf1p at Thr^210^, we determined the cellular content of phospho-Snf1p in osmotin- or adiponectin-treated cells of strain BWG1-7a that were expressing *AdipoR1* or *AdipoR2* constitutively from a multicopy plasmid (Figure S7). No significant increase in phospho-Snf1p levels were observed in empty vector, pAdipoR1 or pAdipoR2 transformants after osmotin or adiponectin treatments. This suggested that IZH2 could be interfering with AdipoR1-dependent Snf1-activation signaling, just as it interferes with APPL1-dependent AdipoR1signaling (Figure 4C). Therefore we repeated this experiment using an *izh2Δ* mutant of strain BWG1-7a. We found the cellular content of Thr^210^-phosphorylated Snf1p was unaffected by osmotin or adiponectin treatments in an *izh2* mutant transformed with the empty vector (Figure 7). Constitutive expression of *AdipoR1* or *AdipoR2* from the same multicopy vector resulted in a time- and concentration-dependent increase in Snf1p phosphorylation at Thr^210^ in response to osmotin or adiponectin treatments (Figures 7 and S8). These results demonstrated that AdipoR1 and AdipoR2 signaling in yeast and mammalian cells share an important common element, namely, the activation of AMPKα by phosphorylation. They also showed that IZH2 interferes with the AdipoR signaling that leads to Snf1p activation in *S. cerevisiae*.

### Osmotin-AdipoR Signaling Results in Suppression of Gene Expression *via* Stress Responsive Promoter Elements

**Figure 8:**
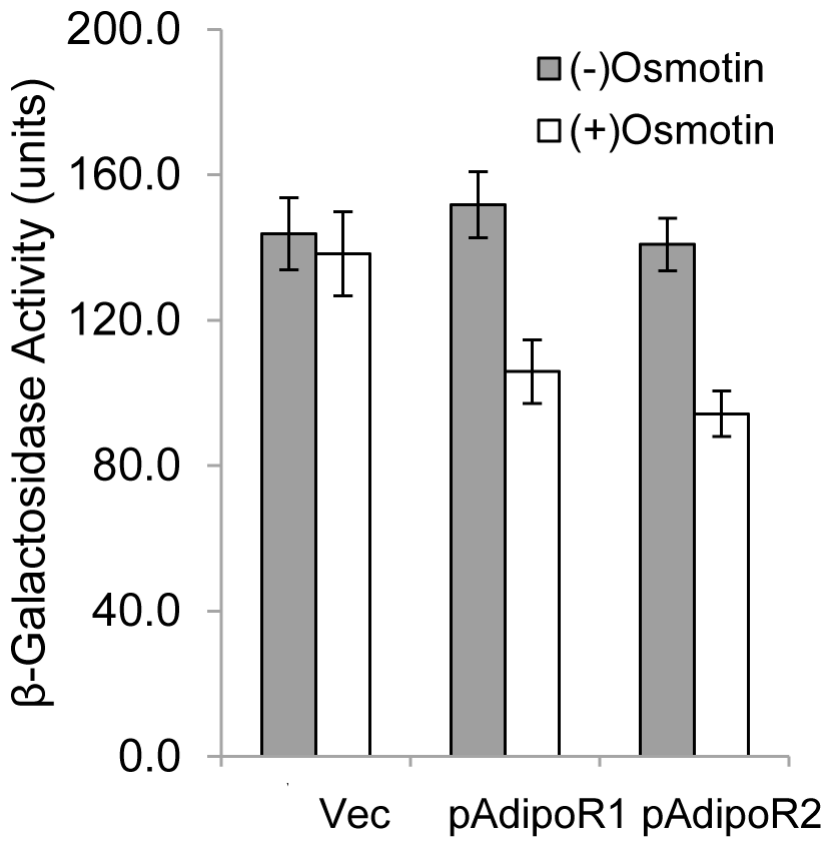
Osmotin signaling mediated by adiponectin receptors induces suppression of gene expression *via* stress responsive promoter elements. Cells (about 10^8^/mL) of strain BWG1-7a *Δizh2::Kan_MX* co-transformed with pSTRE-*lacZ*(*LEU2*) and p426-GPD (Vec), p426-GPD-*AdipoR1* (pAdipoR1) or p426-GPD-*AdipoR2* (pAdipoR2) were treated with osmotin (4 µM) in YPD for 45 min at 30°C and β-galactosidase activity was measured at the end of the treatment period. The values represent the means ± SE of two independent experiments with 3 to 4 samples per experiment.

*IZH2*-mediated osmotin signaling activates the PKA pathway resulting in suppression of transcription from stress responsive promoter elements (STRE, 5′-CCCCT-3′). Mutation of *IZH2* abrogates this effect [29] and conversely, overexpression of *IZH2* suppresses transcription from STREs [26]. Accordingly, we observed that osmotin was unable to suppress STRE-*lacZ* expression in an *izh2Δ* mutant co-transformed with pSTRE-*lacZ*(*LEU*) and p426-GPD vector (Figure 8). However, in an *izh2Δ* mutant co-transformed with pSTRE-*lacZ*(*LEU*) and p426-GPD-*AdipoR1* or p426-GPD- *AdipoR2*, STRE-*lacZ* expression was suppressed by osmotin treatment. Therefore *AdipoR1* and *AdipoR2* resemble *IZH2* in their ability to activate ligand-dependent signaling *via* the PKA pathway in *S. cerevisiae*.

## Discussion

We describe here a *S. cerevisiae* based system for investigating ligand/AdipoR1 interactions that lead to cellular signaling. The three-parts system consists of a split luciferase assay for reporting ligand-AdipoR1 interactions and assays to verify ligand-AdipoR mediated activation of Snf1p (yeast AMPKα) phosphorylation and PKA signaling pathways. Since adiponectin-AdipoR dependent activation of AMPKα phosphorylation and PKA signaling mediate anti-diabetic and cardioprotective effects of adiponectin, our *S. cerevisiae* based system has the potential to facilitate the discovery of AdipoR ligands with therapeutic value.

Besides osmotin and OSMpep [50], there are no known ligands of AdipoR1. Since osmotin and plant TLPs have similar structure and biological activity, and OSMpep is quite conserved between plant TLPs, we performed a comparative analysis of the binding of several homologs of OSMpep to membrane embedded AdipoR1 and were able to validate the ability of the split luciferase assay to discriminate between ligands based on the strength of their binding to AdipoR1 (Figure 6 and Table 1). It should be noted that two biologically active adiponectin peptides that have been described [59], [60] encompass residues that are predicted to interact with AdipoR1 by our model and by the earlier model (Figure S5) [50].

Split luciferase complementation assays have been successfully used to study protein-protein interactions in mammalian and plant systems but are used less frequently in *S. cerevisiae.* Our results show that they can be used to demonstrate protein-protein interactions in whole colonies of *S. cerevisiae* as well as in cells in liquid culture. We decided to base our assay for investigating ligand-AdipoR1 interactions on the first protein-protein interaction of AdipoR1 that is known to trigger downstream signaling underlying the anti-diabetic and cardioprotective effects of adiponectin, *i.e.* the interaction between AdipoRs and the coupling protein APPL1 (Figure 1) [43], [53]. The split luciferase assay could possibly be optimized further by controlling relative expression levels of AdipoR1 and APPL1, for example, by expressing them from promoters regulated by different stimuli. In fact when the signal strengths in response to adiponectin are compared in 1% galactose-grown *S. cerevisiae* cells transformed with pESC-URA-CLuc-AdipoR1-APPL1-NLuc, in which CLuc-AdipoR1 and APPL1-NLuc are expressed under the inducible *GAL1* and *GAL10* promoters on the high copy number p-ESC-URA vector (Figure 5) and in *S.cerevisiae* cells co-transformed with pESC-URA-CLuc-AdipoR1 and p425-GPD-APPL1-NLuc where the only difference is that APPL1-NLuc is expressed from the constitutive GPD promoter on the high copy number p425-GPD vector (Figure S9), the response to adiponectin is greater in the latter instance. Our preliminary results suggest that the split luciferase assay can be adopted for reporting ligand-AdipoR2 interactions (Figure S9). Therefore, with optimization and adaptation to robotic plating and washing, the split luciferase assay has the potential to be useful for screening chemical libraries for candidate AdipoR ligands.

APPL1 is a 709 amino acids protein that - ordered from the N- to the C-terminus - consists of BAR (Bin/Amphiphysin/Rvs), PH (plekstrin homology), BPP (between PH and PTB), PTB (phosphotyrosine binding) and CC (coiled coil) domains [54]. Sip3p is the closest structural relative of APPL1 in *S. cerevisiae*, consisting of 1229 amino acids and having only a PH domain (amino acids 310–423) in common with APPL1. Sip3p was identified as a transcriptional activator that interacts with DNA-bound Snf1p [55]. Inactivation of *SIP3* abrogates *IZH2*- and *AdipoR*-dependent repression of *FET3* promoter activity in *S. cerevisiae* [26] (Figure 1). These data, and the fact that the PH domain of APPL1 which is known to mediate AMPK phosphorylation in mammalian cells [43] is conserved in Sip3p, led us to consider the possibility that Sip3p could interfere with the AdipoR1-APPL1 interaction. However, our results show that Sip3p does not interfere with the APPL1-AdipoR1 interaction (Figure 4C). This is not surprising, since the overall homology between APPL1 and Sip3p is very low and Sip3p also lacks the PTB domain that mediates AdipoR1-APPL1 interaction. Overall, our results suggest that while Sip3p participates in the Snf1p pathway leading to transcriptional regulation of *SUC2* (invertase) and in the pathway leading from *IZH2* or *AdipoRs* to transcriptional regulation of *FET3* [26], [55], it not does not compete with APPL1 for interacting with AdipoR1. APPL1 is required for adiponectin mediated AMPKα activation in mammalian cells [43], but was not required for adiponectin-AdipoR-mediated Snf1p activation in *S. cerevisiae* (Figure 7). Further experiments are necessary to clarify whether Sip3p or some *S. cerevisiae* protein(s) other than Sip3p substitutes for APPL1 function in the pathway from AdipoRs to Snf1 phosphorylation in yeast or whether an APPL1-like function is not required in this pathway.

Among the *S. cerevisiae* IZH family proteins, Izh2p is the most closely related member to AdipoRs (about 32% amino acid sequence identity) with Izh1p being next (about 29% amino acid sequence identity). Izh3p and Izh4p have less than 15% amino acid sequence identity with AdipoRs. Genetic evidence suggests that the AdipoRs as well as the *IZH* family members, *IZH1*, *IZH2*, *IZH3* and *IZH4*, function in the same signaling pathway that leads to transcriptional regulation of *FET3* in *S. cerevisiae* [26]. Accordingly, inactivation of *IZH2* increased the signal in the split luciferase assay of AdipoR1-APPL1 interaction (Figure 4C) indicating that Izh2p competes with AdipoR1 for APPL1. Indeed, we were able to directly verify Izh2p/APPL1 interaction by demonstrating that luciferase activity increased as a function of galactose concentration in the growth medium for cells of strain BY4741 co-transformed with pESC-URA- CLuc-IZH2 and p425GPD-APPL1-NLuc (Figure S9C). *IZH2* interfered with signaling leading from AdipoRs to the activation of Snf1p phosphorylation (compare Figures 7 and S7). It is therefore possible that the signal strength or/and specificity for AdipoR1 in our *S. cerevisiae* based assays for ligand-AdipoR1 interactions could be further improved by performing experiments in a yeast strain lacking both *IZH2* and *IZH1* or all four *IZH* genes.

In the *izh2* mutant expressing AdipoR1, both osmotin and adiponectin were able to activate Snf1p phosphorylation. On the other hand, in the *izh2* mutant expressing AdipoR2 only adiponectin was able to activate Snf1p phosphorylation. Our previous results have indicated that AdipoR1 or AdipoR2 contribute equally to stimulation of AMPK phosphorylation in response to osmotin and adiponectin treatments in the immortalized murine C2C12 myogenic cell line [29]. Also both AdipoR1 and AdipoR2 appear to be equally functional in the pathway leading from osmotin to STRE-*lacZ* expression (Figure 8). So how does one explain the inability of osmotin-AdipoR2 to stimulate Snf1p phosphorylation in *S. cerevisiae*? First, the apparent equal contribution of AdipoR1 and AdipoR2 to adiponectin and osmotin mediated AMPK phosphorylation in the C2C12 immortalized mouse myoblast cell line and STRE-*lacZ* expression in yeast must be viewed with caution because only one concentration of each agonist was used in these experiments. Second, it is important to recognize here that several factors contribute to the nature and amplitude of signaling output from adiponectin [20]. These include the molecular form (globular, full length, glycosylated full length) of adiponectin, the relative proportion of AdipoR1 and AdipoR2 in the plasma membrane, the relative abundance of different downstream signaling components in different cell types, differences in the affinities of the two receptors for a given intracellular signaling molecule and differences in signaling networks in different cell types. Therefore it is very difficult to extrapolate results from one set of conditions to another. However, we can provide one explanation to reconcile the apparent discrepancies between the ability of AdipoR1 and AdipoR2 to contribute to osmotin-induced Snf1p phosphorylation in *S. cervisiae* (Figure 7) and C2C12 myocytes [29] based on the known differences in the affinities of AdipoRs for different molecular forms of adiponectin [21]. AdipoR1 binds to globular adiponectin with high affinity and to bacterially expressed full length adiponectin with low affinity whereas AdipoR2 binds to both these forms of adiponectin with medium affinity [21]. We used bacterially expressed full length adiponectin in all of the experiments in this study. If we postulate that the strength of Snf1p/AMPKα phosphorylation is governed solely by the binding affinity of ligand for AdipoRs, we can expect somewhat comparable effects for the induction of Snf1p phosphorylation by full length adiponectin-AdipoR1and full length adiponectin-AdipoR2 in *S. cerevisiae*, as observed (Figure 7). Osmotin and globular adiponectin lack the N-terminal collagen domain of full length adiponectin and therefore do not exist as hexamers like full length adiponectin. Therefore the affinity of osmotin for AdipoR1 would exceed its affinity to AdipoR2, as is the case for globular adiponectin, resulting in significantly greater osmotin-induced Snf1p phosphorylation in *S. cerevisiae* cells expressing AdipoR1 than in cells expressing AdipoR2 (Figure 7). On the other hand, we used globular adiponectin and osmotin in our previous study [29] and observed, accordingly, that silencing of either AdipoR1 or AdipoR2 had the same effect on osmotin- and adiponectin-induced AMPKα phosphorylation. The discrepancy between the ability of AdipoR1 and AdipoR2 to contribute to osmotin-induced Snf1p phosphorylation in *S. cervisiae* (Figure 7) and C2C12 myocytes [29] can also be explained in another way. Osmotin stimulates *IZH2*-dependent and *IZH2*-independent signaling in S. cerevisiae [29] and therefore stimulates AdipoR-independent pathways also. If we assume that an AdipoR-independent pathway(s) specifically and strongly inhibits the AdipoR2 dependent Snf1p phosphorylation, it will appear as though osmotin signaling is connected to AdipoR1 not AdipoR2 in *S. cerevisiae*. If osmotin fails to stimulate an AdipoR-independent pathway that inhibits AMPKα phosphorylation in the mammalian cells, it would appear as if both AdipoR1 and AdipoR2 contribute to osmotin activated AMPKα phosphorylation in the mammalian cells. There are possibly several other explanations for the discrepancy between the ability of AdipoR1 and AdipoR2 to contribute to osmotin-induced Snf1p phosphorylation in *S. cerevisiae* (Figure 7) and C2C12 myocytes [29]. Clearly our cell signaling assays can be improved when connections leading from ligand-AdipoRs to Snf1p phosphorylation and STRE-*lacZ* expression in *S. cerevisiae* are elucidated further.

Besides its use in the identification of new agonists of AdipoRs, the *S.cerevisiae* system described herein has potential for substantially increasing our understanding of ligand-AdipoR interactions and the resultant downstream signaling. For example, the spilt luciferase system can be used to screen a library of receptor mutants to delineate residues important for AdipoR-APPL1 interaction and AdipoR-ligand interactions. It should also be possible to identify residues of APPL1 that are necessary for AdipoR-APPL1 interaction by a similar approach. By screening a collection of *S. cerevisiae* mutants for inability to support the AdipoR1-APPL1 interaction, proteins required to stabilize this interaction can be discovered. Similarly, by transforming an expression library of mammalian cDNAs into *S. cerevisiae* having the CLuc-AdipoR/APPL1-NLuc constructs, it should be possible to identify mammalian proteins involved in stabilization/destabilization of the AdipoR-APPL1 complex. All of these studies will be greatly facilitated by our observation that the AdipoR-APPL1 interaction can be scored in *S. cerevisiae* colonies on plates. The basic understanding required to identify new pharmaceutically valid targets and the identification of AdipoR ligands that can be facilitated by our *S. cerevisiae* assay system should have considerable value in discovery of therapeutic interventions for metabolic disorders.

## Supporting Information

Figure S1 **The AdipoR1 ligands, adiponectin and osmotin, induce increase in luciferase reporter activity.** Cells of strain BY4741carrying pESC-URA-CLuc-AdipoR1-APPL1-NLuc were grown for 16 h at 30°C in selective minimal medium at the indicated galactose concentrations, treated for 4 h at 30°C with the indicated test compounds and then assayed for luciferase activity. A representative image of relative luciferase activity with the different test compounds is shown for cells grown on 1% and 1.5% galactose. Luciferase (Luc) activities represent the means ± SD from three independent experiments with triplicate samples Symbols: PBS, 1/8 X PBS; OSM, 6.4 µM osmotin; f-ADPN, 2.8 µM bacterially expressed full length adiponectin; g-ADPN, 2.4 µM bacterially expressed globular adiponectin; f-ADPN*, 1.3 µM bacterially expressed full length adiponectin from a commercial source.
(TIF)

Figure S2 (A) Homology model of AdipoR1 embedded in membrane structure in preparation for molecular dynamics simulations. AdipoR1 is shown with orange color; DPPC membranes are shown with cyan color while water molecules are shown with O-red color and H-white color. (B) RMSD vs time after 50 ns of MD simulation at 300 K. The red curve indicates RMSD for Cα atoms and the black curve shows the RMSD for backbone atoms.
(TIF)

Figure S3 **3D models of proteins and protein complexes used in this study.** (A) Homology model of AdipoR1. Residues 355–374 are marked in yellow. These residues are not considered in the homology model developed by [49]. (B) Homology 3D-model of trimeric human globular adiponectin. The beta strands are indicated by arrows. Bound Ca^2+^ ions are shown as yellow spheres. (C) 3D-structure of osmotin (PDB id. 1PCV-chain A). (D) 3D model of the AdipoR1 binding site. Common residues of AdipoR1 interacting with both, adiponectin and osmotin are shown in blue. Residues interacting with adiponectin only are shown in red. (E) 3D model of the AdipoR1/adiponectin complex. (F) 3D model of AdipoR1/osmotin complex. (G) Superposition of homology model of adiponectin (cyan) and crystal structure of adiponectin trimer (PDB id: 4DOU; orange). The RMSD between Cα atoms is 0.45 Å. Close-up shows loops of adiponectin involved with interaction with AdipoR1.
(TIF)

Figure S4 **Representation of AdipoR1 residues that interact with ligands used in this study.** Interacting residues of AdipoR1 in the top-scoring complexes with adiponectin (ADPN), osmotin and the TLP peptides shown in Table 1 are shown. Interacting residues that were predicted by our model are shown in red font and interacting residues predicted by the model of [49] are shown in green font. C-terminal residues of AdipoR1 not included into the models are shown in gray font. P1, P2 and P3 represent the interactions of AdipoR1 with adiponectin and osmotin in the first, second and third top-scoring complexes.
(TIF)

Figure S5 **Complete sequences of protein models used in this study.** Shown in gray font are residues of the deduced amino acid sequences of all three proteins that were not used in the model. These include the N-terminal signal sequence of all the proteins, the intracellular N-terminal sequence of AdipoR1, the collagen domain of adiponectin and the C-terminal vacuolar targeting sequence of osmotin. Amino acids indicated by the red font represent the residues of AdipoR1 that interact with either adiponectin or osmotin, residues of adiponectin that interact with AdipoR1 and residues of osmotin that interact with AdipoR1, respectively. The OSM_pep_ fragment (residues 157–165) is underlined and shown in bold.
(TIF)

Figure S6 **Comparison of the binding of osmotin and OSMpep to AdipoR1.** (A) Overlay of the three top scoring OSMpep poses (Green = best, purple = second, orange = third). (B) Overlay of the three top scoring OSMpep poses and osmotin (red).
(TIF)

Figure S7 **Osmotin signaling mediated by adiponectin receptors does not induce Snf1p phosphorylation in wild type BWG1-7a cells.** Cells (about 10^8^/mL) of strain BWG1-7a transformed with p426GPD (Vec), p426GPD-*AdipoR1* (pAdipoR1) or p426GPD-*AdipoR2* (pAdipoR2) were treated with osmotin and adiponectin at 30°C in YPD. Aliquots were withdrawn for viable counts determination before the cell lysates were prepared for analysis by10% SDS-PAGE. 100 µg total proteins were loaded per lane. (A) Time dependent changes in Snf1p phosphorylation with osmotin (0.5 µM) or adiponectin (0.5 µM). Shown are blots probed first with phospho-AMPK(Thr-172) antibody, then stripped and probed with actin antibody. (B,C) Osmotin and adiponectin concentration dependent changes in Snf1p pshosphorylation. Treatment time was 30 min. Shown in the top panels are blots probed first with phospho-AMPK(Thr-172) antibody, then stripped and probed with actin antibody. Shown in the middle panels are relative band intensities in the depicted gels. ‘Relative band intensity’ was defined as the ratio of the intensity of phospho-Snf1p signal to actin signal for each lane when the value of this ratio for the corresponding untreated Vec sample was arbitrarily assigned the value 1.0. Shown in the bottom panels are viable counts in each sample at the end of the treatments. The experiments were performed twice with comparable results and the results of one experiment are shown.
(TIF)

Figure S8 **Time dependent increase in Snf1 phosphorylation in** ***izh2*** **mutant in response to osmotin or adiponectin treatment.** Cells (about 10^8^/mL) of strain BWG1-7a *izh2Δ* transformed with p426GPD (Vec), p426GPD-*AdipoR1* (pAdipoR1) or p426GPD-*AdipoR2* (pAdipoR2) were treated with osmotin (0.5 µM) or adiponectin (0.5 µM) at 30°C in YPD for the indicated times. Shown are blots of total protein extracts fractionated by 10% SDS-PAGE that were probed first with phospho-AMPK(Thr-172) antibody, then stripped and probed with actin antibody. 100 µg total proteins were loaded per lane.
(TIF)

Figure S9 **Luciferase reporter activity depends on the expression level of receptor at a constant level of APPL1.** (A) RT-PCR analysis of *AdipoR1* and *APPL1* expression in total RNA (2 µg) from cells of strain BY4741 carrying indicated plasmids that were grown for 16 h at 30°C in selective minimal medium containing 2% galactose. *ACT1* expression is shown for normalization. (B-D) Quantification of luciferase activity in BY4741 cells carrying indicated plasmids. Cells were grown for 16 h at 30°C in selective minimal medium at the indicated galactose concentrations. The final concentration of sugars in all media was adjusted to 2% with raffinose. Shown is quantification of luciferase activity. Inset is a representative image of the microplate (showing from left, cells grown in 0, 0.25, 0.5, 1, 1.5, and 2% galactose). (E) Adiponectin stimulates luciferase activity. Cells of strain BY4741 carrying p425GPD-APPL1-NLuc and either pESC-URA-CLuc-AdipoR1 or pESC-URA-CLuc-AdipoR2 were grown for 16 h at 30°C in selective minimal medium at the indicated galactose concentrations, treated for 2 h at 30°C with 1/8 X PBS (−) or full length bacterially expressed adiponectin (+). Luciferase activity was then visualized by imaging.
(TIF)

Table S1 **Primers used for construction of plasmids.**
(DOCX)

Table S2 **Primers used for gene expression analysis by RT-PCR.**
(DOCX)

Methods S1 Click here for additional data file. (DOCX)
