# Supplementary figures and images for: A Saccharomyces cerevisiae Assay System to Investigate Ligand/AdipoR1 Interactions That Lead to Cellular Signaling
Source: PLoS One. 2013 Jun 7;8(6):e65454. doi: 10.1371/journal.pone.0065454 (PMC3676391; doi:10.1371/journal.pone.0065454)

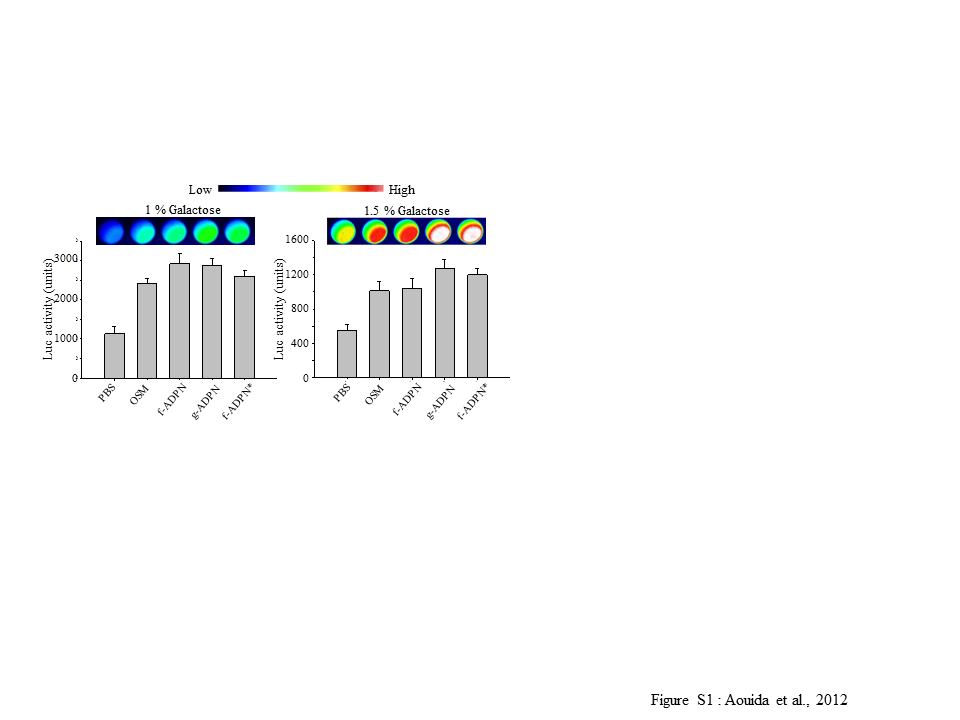

Supplement: Figure S1 — The AdipoR1 ligands, adiponectin and osmotin, induce increase in luciferase reporter activity. Cells of strain BY4741carrying pESC-URA-CLuc-AdipoR1-APPL1-NLuc were grown for 16 h at 30°C in selective minimal medium at the indicated galactose concentrations, treated for 4 h at 30°C with the indicated test compounds and then assayed for luciferase activity. A representative image of relative luciferase activity with the different test compounds is shown for cells grown on 1% and 1.5% galactose. Luciferase (Luc) activities represent the means ± SD from three independent experiments with triplicate samples Symbols: PBS, 1/8 X PBS; OSM, 6.4 µM osmotin; f-ADPN, 2.8 µM bacterially expressed full length adiponectin; g-ADPN, 2.4 µM bacterially expressed globular adiponectin; f-ADPN*, 1.3 µM bacterially expressed full length adiponectin from a commercial source. (TIF) [file pone.0065454.s001.tif]

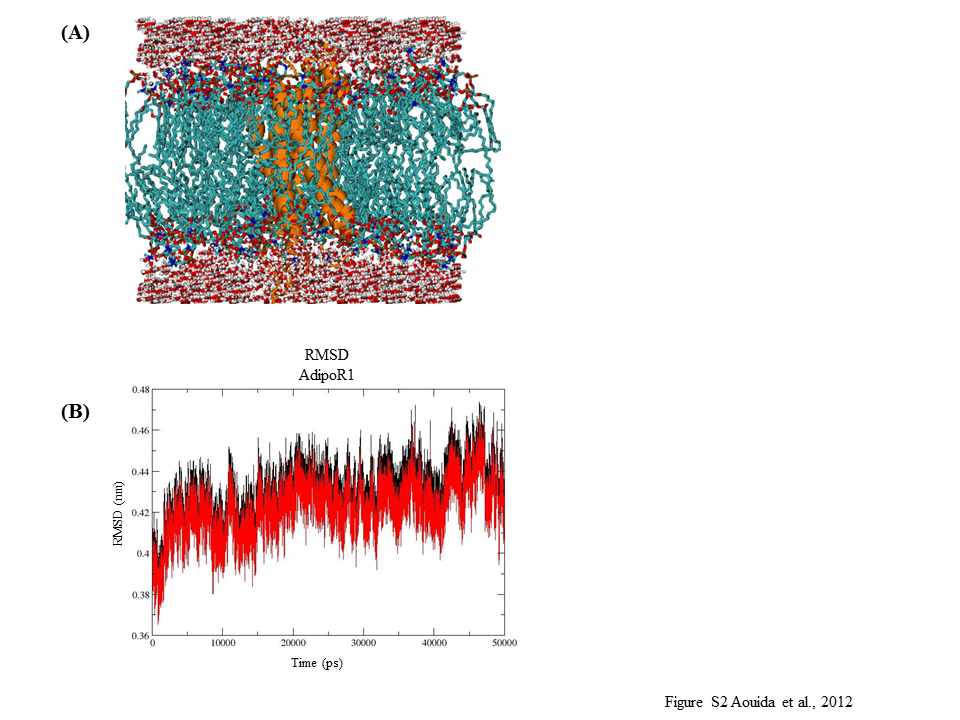

Supplement: Figure S2 — (A) Homology model of AdipoR1 embedded in membrane structure in preparation for molecular dynamics simulations. AdipoR1 is shown with orange color; DPPC membranes are shown with cyan color while water molecules are shown with O-red color and H-white color. (B) RMSD vs time after 50 ns of MD simulation at 300 K. The red curve indicates RMSD for Cα atoms and the black curve shows the RMSD for backbone atoms. (TIF) [file pone.0065454.s002.tif]

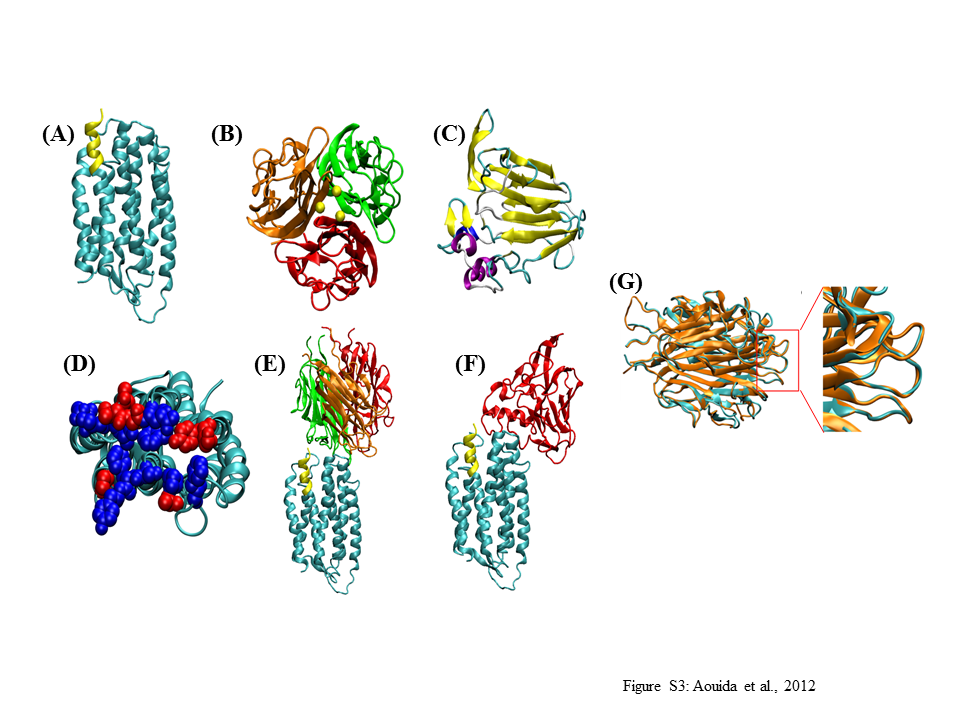

Supplement: Figure S3 — 3D models of proteins and protein complexes used in this study. (A) Homology model of AdipoR1. Residues 355–374 are marked in yellow. These residues are not considered in the homology model developed by [49]. (B) Homology 3D-model of trimeric human globular adiponectin. The beta strands are indicated by arrows. Bound Ca2+ ions are shown as yellow spheres. (C) 3D-structure of osmotin (PDB id. 1PCV-chain A). (D) 3D model of the AdipoR1 binding site. Common residues of AdipoR1 interacting with both, adiponectin and osmotin are shown in blue. Residues interacting with adiponectin only are shown in red. (E) 3D model of the AdipoR1/adiponectin complex. (F) 3D model of AdipoR1/osmotin complex. (G) Superposition of homology model of adiponectin (cyan) and crystal structure of adiponectin trimer (PDB id: 4DOU; orange). The RMSD between Cα atoms is 0.45 Å. Close-up shows loops of adiponectin involved with interaction with AdipoR1. (TIF) [file pone.0065454.s003.tif]

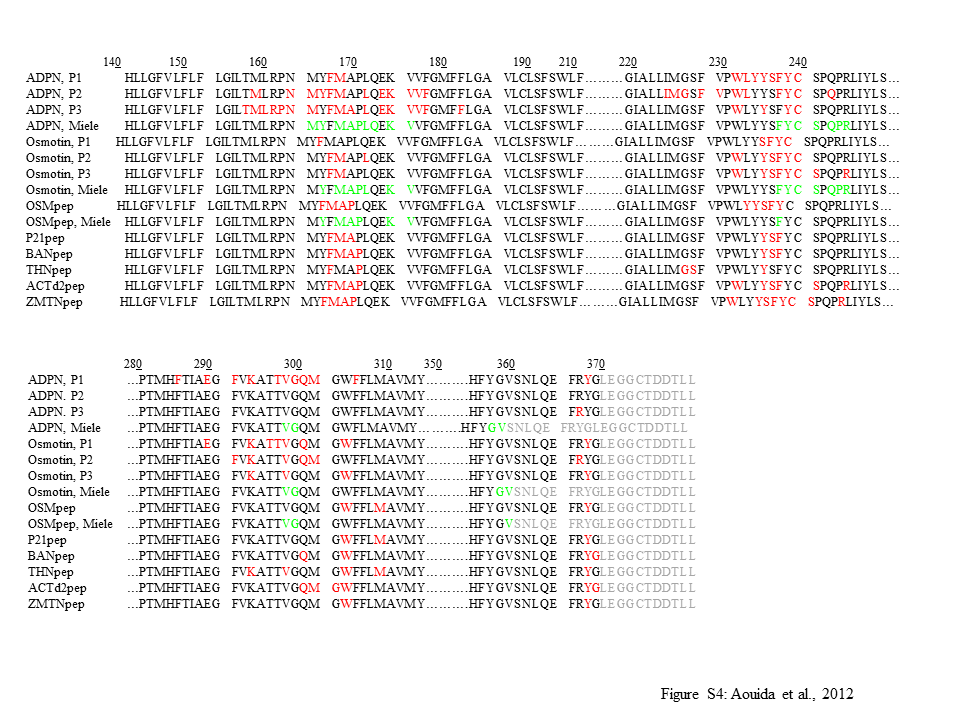

Supplement: Figure S4 — Representation of AdipoR1 residues that interact with ligands used in this study. Interacting residues of AdipoR1 in the top-scoring complexes with adiponectin (ADPN), osmotin and the TLP peptides shown in Table 1 are shown. Interacting residues that were predicted by our model are shown in red font and interacting residues predicted by the model of [49] are shown in green font. C-terminal residues of AdipoR1 not included into the models are shown in gray font. P1, P2 and P3 represent the interactions of AdipoR1 with adiponectin and osmotin in the first, second and third top-scoring complexes. (TIF) [file pone.0065454.s004.tif]

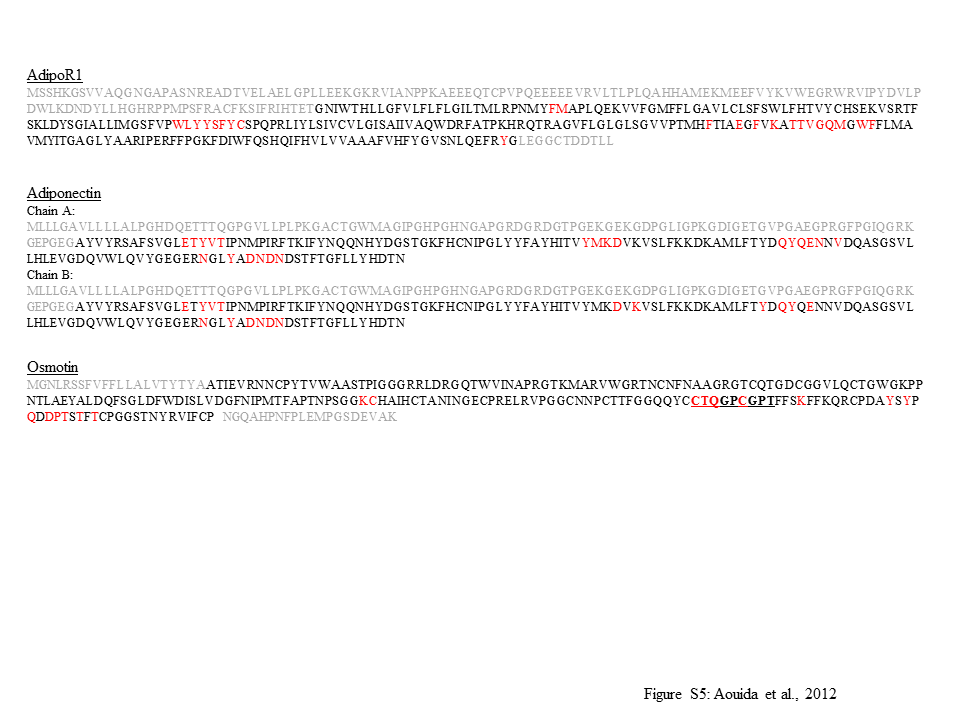

Supplement: Figure S5 — Complete sequences of protein models used in this study. Shown in gray font are residues of the deduced amino acid sequences of all three proteins that were not used in the model. These include the N-terminal signal sequence of all the proteins, the intracellular N-terminal sequence of AdipoR1, the collagen domain of adiponectin and the C-terminal vacuolar targeting sequence of osmotin. Amino acids indicated by the red font represent the residues of AdipoR1 that interact with either adiponectin or osmotin, residues of adiponectin that interact with AdipoR1 and residues of osmotin that interact with AdipoR1, respectively. The OSMpep fragment (residues 157–165) is underlined and shown in bold. (TIF) [file pone.0065454.s005.tif]

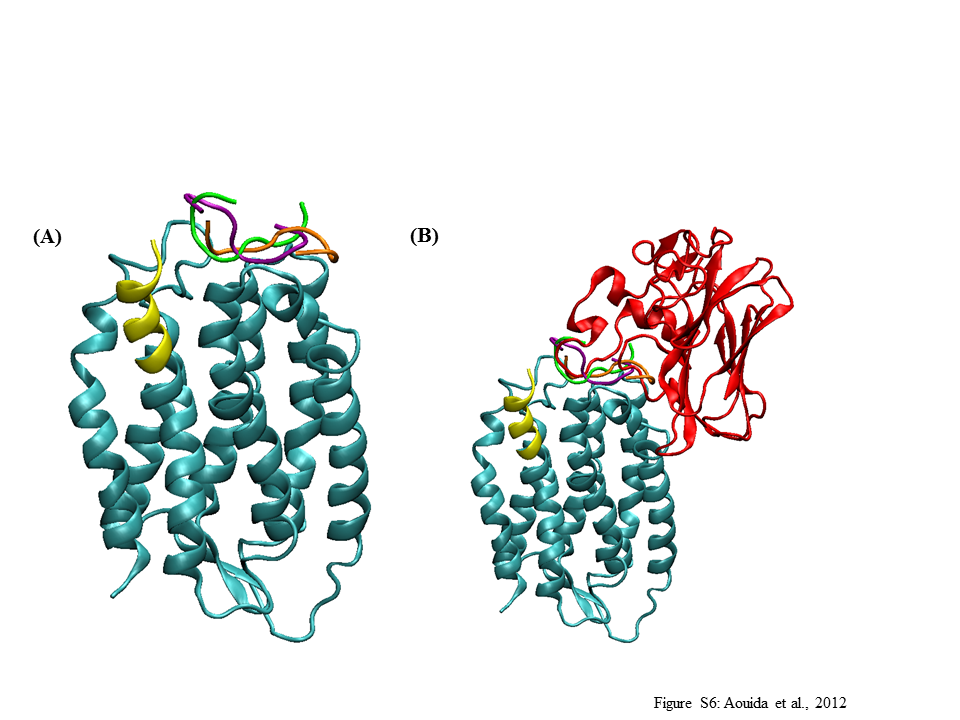

Supplement: Figure S6 — Comparison of the binding of osmotin and OSMpep to AdipoR1. (A) Overlay of the three top scoring OSMpep poses (Green = best, purple = second, orange = third). (B) Overlay of the three top scoring OSMpep poses and osmotin (red). (TIF) [file pone.0065454.s006.tif]

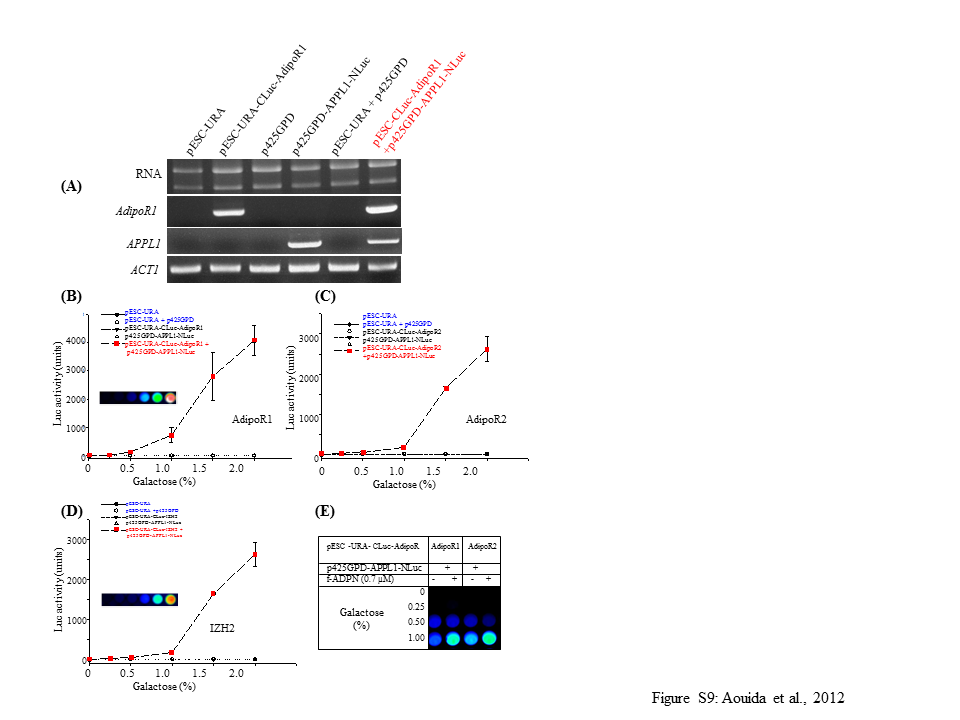

Supplement: Figure S9 — Luciferase reporter activity depends on the expression level of receptor at a constant level of APPL1. (A) RT-PCR analysis of AdipoR1 and APPL1 expression in total RNA (2 µg) from cells of strain BY4741 carrying indicated plasmids that were grown for 16 h at 30°C in selective minimal medium containing 2% galactose. ACT1 expression is shown for normalization. (B-D) Quantification of luciferase activity in BY4741 cells carrying indicated plasmids. Cells were grown for 16 h at 30°C in selective minimal medium at the indicated galactose concentrations. The final concentration of sugars in all media was adjusted to 2% with raffinose. Shown is quantification of luciferase activity. Inset is a representative image of the microplate (showing from left, cells grown in 0, 0.25, 0.5, 1, 1.5, and 2% galactose). (E) Adiponectin stimulates luciferase activity. Cells of strain BY4741 carrying p425GPD-APPL1-NLuc and either pESC-URA-CLuc-AdipoR1 or pESC-URA-CLuc-AdipoR2 were grown for 16 h at 30°C in selective minimal medium at the indicated galactose concentrations, treated for 2 h at 30°C with 1/8 X PBS (−) or full length bacterially expressed adiponectin (+). Luciferase activity was then visualized by imaging. (TIF) [file pone.0065454.s009.tif]
