# Supplementary material for: A Saccharomyces cerevisiae Assay System to Investigate Ligand/AdipoR1 Interactions That Lead to Cellular Signaling
Source: PLoS One. 2013 Jun 7;8(6):e65454. doi: 10.1371/journal.pone.0065454 (PMC3676391; doi:10.1371/journal.pone.0065454)
Supplement: Table S1 — Primers used for construction of plasmids. (DOCX) [file pone.0065454.s010.docx]

**Table S1:** Primers used for construction of plasmids.

| Primer name | Sequence (5’-…..-3’) |
| --- | --- |
| AdipoR1-F-Spe1 | ACTAGTATGTCTTCCCACAAAGGA |
| AdipoR1-R-Cla1 | ATCGATTCAGAGAAGGGTGTCATC |
| AdipoR2-F-Spe1 | ACTAGTATGAACGAGCCAACAGAA |
| AdipoR2-R-Cla1 | ATCGATTCACAGTGCATCCTCTTC |
| APPL1-F-KpnI-SpeI | TGTAGGTACCACTAGTATGCCGGGGATCGACAAGCTGCCCAT |
| APPL1-R-SalI-1 | TGTAGTCGACTGCTTCTGATTCTCTCTTCTTTCC |
| CLuc-F-BamHI | TGTGGGATCCATGTCCGGTTATGTAAACAATC |
| CLuc-R- HindIII-AscI- XhoI | TGTGAAGCTTGGCGCGCCCTCGAGGCCCCGGGACGCGTACGAG |
| AdipoR1-F-XhoI | TGTGCTCGAGATGTCTTCCCACAAAGGATC |
| AdipoR1-HindIII | TGTGCTCGAGATGTCTTCCCACAAAGGATC |
| AdipoR2-F-XhoI | TGTGCTCGAGATGAACGAGCCAACAGAAAACCG |
| AdipoR2-HindIII | TGTGAAGCTTTCACAGTGCATCCTCTTCAC |
| APPL1-F-NotI | TATAGCGGCCGCATGCCGGGGATCGACAAGCT |
| NLuc-R-ClaI | TGTGATCGATTCATCCATCCTTGTCAATCAAG |
| GFP-AdipoR1 F-EcoR1 | GCTGCTGGGATTACACATGGCATGGATGAACTATACAAAGAATTCATGTCTTCCCACAAAGGATC |
| GFP-AdipoR1-R-Xho1 | CTGTATTGATTAATGTACTACGCCGGGAGATCTACGTACGAGCTCTCAGAGAAGGGTGTCATCAG |
| GFP-AdipoR2-F-EcoR1 | GCTGCTGGGATTACACATGGCATGGATGAACTATACAAAGAATTCATGAACGAGCCAACAGAAAA |
| GFP-AdipoR2-R-Xho1 | CTGTATTGATTAATGTACTACGCCGGGAGATCTACGTACGAGCTCTCACAGTGCATCCTCTTCAC |

Restriction sites are underlined
