# Supplementary material for: A Saccharomyces cerevisiae Assay System to Investigate Ligand/AdipoR1 Interactions That Lead to Cellular Signaling
Source: PLoS One. 2013 Jun 7;8(6):e65454. doi: 10.1371/journal.pone.0065454 (PMC3676391; doi:10.1371/journal.pone.0065454)
Supplement: Table S2 — Primers used for gene expression analysis by RT-PCR. (DOCX) [file pone.0065454.s011.docx]

**Table S2:** Primers used for gene expression analysis by RT-PCR.

| Primer Name | Sequence (5’-…..-3’) |
| --- | --- |
| RT-PCR AdipoR1-F | AGTGCCCCAGGAAGAAGAGGAGGAGGTGCGGGTACT |
| RT-PCR-AdipoR1-R | TGGAGAGGTAGATGAGCCGTGGCTGTGGGGAGCAGT |
| RT-PCR-AdipoR2-F | GGTATGGGAAGGTCGGTGGCGAGTGATCCCTCATGA |
| RT-PCR-AdipoR2-R | TCCCACTGGGAGACTATAATGGCTGCAATGCCCAGC |
| RT-PCR-APPL1-F | AGCCAGTGATCCCTTATATGTGCCTGACCCAGACCC |
| RT-PCR-APPL1-R | TCCTAAGGATCCTGAACTGCTGGTTCGAGCTGTCGG |
| RT-PCR-ACT1-F | GAGTGTGCAAGAGCTGAAGAAGAGAGCTGCAGGGAA |
| RT-PCR-ACT1-R | ACTACACGCGATCCCAAAGCTAACGGTAATAAGCGC |
