# Supplementary material for: A Saccharomyces cerevisiae Assay System to Investigate Ligand/AdipoR1 Interactions That Lead to Cellular Signaling
Source: PLoS One. 2013 Jun 7;8(6):e65454. doi: 10.1371/journal.pone.0065454 (PMC3676391; doi:10.1371/journal.pone.0065454)
Supplement: Methods S1 [file pone.0065454.s012.docx]

**Supplemental Methods**

Homology Modeling for Human AdipoR1

The 3D-homology model covering amino acids 135 - 364 of the human adiponectin receptor 1 (AdipoR1), (UniProt id: Q96A54) was built using Prime module of Schrödinger suite (Prime, version 2.2, Schrödinger, LLC, New York, NY, 2008). In agreement with a previous study [[1](#_ENREF_1)], we found that the structure of archeal (*Natronomonos pharaonis*) phototaxis protein rhodopsin II (PDB id: 1GU8) [[2](#_ENREF_2)] presented the best template among the available transmembrane proteins in PDB database. This template shows only 10 % sequence similarity with human AdipoR1 within the aligned sequence (amino acids 135 – 364 of AdipoR1 and 2- 219 of rhodopsin II) and well resembles the predicted secondary structure elements (arrangement and length of helices and loops) of AdipoR1. ‘Align GPCR’ which is a specially designed program, capable of aligning GPCRs by identifying transmembrane helixes and fingerprint matching was used to suggest the pair-wise alignment between the template and the query sequences. The *build structure screen* was used to predict the homology structure and optimize the side chains of the receptor model. The model was refined to achieve the predicted 7-transmembrane helix architecture. The homology model of AdipoR1 has 91 % residues in favored regions in the Ramchandran plot. The secondary structure predicted by TMpred agreed well with the structure obtained by comparative modeling. The seven transmembrane helices are well-conserved in the secondary structure of AdipoR1 and rhodopsin. The protein preparation wizard was used to add hydrogens to residues, to adjust protonation state, and to cap the terminal amino acids (N-terminus: Ac-HN-, C-terminus: -CO-NH-CH_3_). The resulting AdipoR1 homology model was embedded in a dipalmitoyl phosphatidyl choline (DPPC) membrane layer and molecular dynamics simulation was carried out for 50 ns (see Supplemental Figure S2 and molecular dynamic studies below for details). Our model confirms the overall architecture found by a recent model of Miele et al [[1](#_ENREF_1)].

Homology Modeling of Trimeric Globular Human Adiponectin

The three-dimensional (3D) structure of the globular domain (residues 105-254) of human adiponectin was built using YASARA Structure molecular modeling package (Ver. 11.3.2) [[3](#_ENREF_3)]. The hm_build.mcr macro of the YASARA package with default parameters was used. YASARA identified murine adiponectin as the sole template (PDB id: 1C3H) [[4](#_ENREF_4)]. Amino acids 111 – 247 of this murine adiponectin structure were used as template. The model was subjected to further refinement using the md_refine.mcr macro of YASARA and to 1 ns constant temperature (300 K) and pressure (1 bar) molecular dynamics (MD) simulations using the AMBER03 force field. Simulation parameters were kept at the values defined by the macro. The structure of the protein was simulated in an 8 × 8 × 8 nm rectangular box with periodic boundaries and endcapping with an N-acetyl group to preserve the electronic structure of the backbone.

Molecular Dynamics Studies

MD simulations were performed using the GROMACS 4.5.5 software package [[5](#_ENREF_5)]. First, the homology model of AdipoR1 was embedded in DPCC layer. The GROMOS53a6 force field [[6](#_ENREF_6)] was used to describe the protein part of the model. For the DPPC layer, parameters developed by Tieleman et *al.* were used [[7](#_ENREF_7)]. MD simulation was carried out for 50 ns.

Adiponectin/AdipoR1 and osmotin/AdipoR1 complexes obtained after PATCHDOCK and FireDock analysis (see below) were subjected to MD relaxation. At a pH of 7.0, the system was solvated with TIP3P water molecules and neutralized by adding counter ions. The solvated structures were subject to energy minimization by the steepest descent method. A constrained MD run (protein frozen) was undertaken for 500 ps at 300 K to relax the protein environment. Then, a non-constrained MD run was carried out for 10 ns at 1 bar pressure by coupling the system to external heat and pressure bath. Snapshots of the trajectories were taken at every 2 fs. The first 1 ns point was considered as equilibration period and was not used for subsequent analysis. A reaction-field correction was used for long-range electrostatic interactions, and an energy dispersion correction was implemented. The root-mean-square deviation (RMSD) of the backbone of the protein structure was calculated using the g_rms utility of GROMACS.

Models of Adiponectin/AdipoR1 and Osmotin/AdipoR1 Complexes

Protein-protein docking was carried out using the PatchDock program [[8](#_ENREF_8)]. Previously built homology model for adiponectin, AdipoR1 and crystal structure of osmotin (PDB id: 1PCV, Chain-A) [[9](#_ENREF_9)] was used to build the model for AdipoR1/adiponectin and AdipoR1/osmotin. This program uses a geometry based molecular docking algorithm that yields good molecular complementary shape. Docking is followed by flexible refinement and re-ranking of the rigid docking candidates using FireDock. FireDock accounts for flexibility to the backbone and sidechains. The structures were analyzed using the VMD program [[10](#_ENREF_10)].

Peptide Docking Studies

The peptide ligands were either extracted from available protein structures or built using the Maestro module of the Schrodinger Suite. Ligands were minimized using the Macromodel module of Schrodinger. Minimized peptide ligands were docked to AdipoR1 using the Schrodinger docking program Glide [[11](#_ENREF_11),[12](#_ENREF_12),[13](#_ENREF_13)]. The extracellular surface of AdipoR1 was defined as the active site. In pose prediction studies, a maximum of 15 poses per ligand was saved, while in enrichment studies one pose was saved per ligand. Once initial the Glide docked poses were obtained, Prime side chain and backbone refinement, together with the minimization of the docked pose, were carried out within a sphere of 5 Å from each saved pose.

Analysis interacting residues within AdipoR1/ligand complexes

Intermolecular interactions between protein receptor and ligands were analyzed using Protein Interaction Calculators (PIC) [[14](#_ENREF_14)]. PIC is a server which, given the coordinate set of 3D structure of a protein or an assembly, computes various interactions such as disulphide bonds, interactions between hydrophobic residues, ionic interactions, hydrogen bonds, aromatic– aromatic interactions, aromatic–sulphur interactions and cation–π interactions within a protein or between proteins in a complex.

Evaluation of Models and Sequence Coverage

The AdipoR1 sequence used for the homology model presented by Miele et al [[1](#_ENREF_1)] ends at amino acid 335. This seems reasonable since amino acid 354 is a glycine, which is known to act as a helix breaker. Correspondingly, residues following G354 may form a structurally ill-defined, extracellular cellular domain. However, it is evident form the model of Miele et al [[1](#_ENREF_1)], as well as from our model, that helix-7 does not reach the membrane surface at G354 and correspondingly the following residues are still membrane embedded. We assumed that membrane embedding and helix bundle contacts should result in an extension of helix 7 to the membrane surface. Correspondingly, our AdipoR1 homology model extends until G364, which should terminate the alpha-helical secondary structure due to lack of stabilizing intra membrane environment and contact to the helix bundle. Our MD studies suggest a very stable alpha-helical structure up to residue G364 if AdipoR1 is embedded in the cell membrane. Interactions were generally determined by a distance criterion (< 0.35 nm).

**Supplementary References**

1. Miele M, Costantini S, Colonna G (2011) Structural and functional similarities between osmotin from Nicotiana tabacum seeds and human adiponectin. PLoS One 6: e16690.

2. Edman K, Royant A, Nollert P, Maxwell CA, Pebay-Peyroula E, et al. (2002) Early structural rearrangements in the photocycle of an integral membrane sensory receptor. Structure 10: 473-482.

3. Krieger E, Koraimann G, Vriend G (2002) Increasing the precision of comparative models with YASARA NOVA--a self-parameterizing force field. Proteins 47: 393-402.

4. Berman HM, Westbrook J, Feng Z, Gilliland G, Bhat TN, et al. (2000) The Protein Data Bank. Nucleic Acids Res 28: 235-242.

5. Van Der Spoel D, Lindahl E, Hess B, Groenhof G, Mark AE, et al. (2005) GROMACS: fast, flexible, and free. J Comput Chem 26: 1701-1718.

6. Oostenbrink C, Villa A, Mark AE, van Gunsteren WF (2004) A biomolecular force field based on the free enthalpy of hydration and solvation: the GROMOS force-field parameter sets 53A5 and 53A6. J Comput Chem 25: 1656-1676.

7. Tieleman DP, Marrink SJ, Berendsen HJ (1997) A computer perspective of membranes: molecular dynamics studies of lipid bilayer systems. Biochim Biophys Acta 1331: 235-270.

8. Schneidman-Duhovny D, Inbar Y, Nussinov R, Wolfson HJ (2005) PatchDock and SymmDock: servers for rigid and symmetric docking. Nucleic Acids Res 33: W363-367.

9. Min K, Ha SC, Hasegawa PM, Bressan RA, Yun DJ, et al. (2004) Crystal structure of osmotin, a plant antifungal protein. Proteins 54: 170-173.

10. Humphrey W, Dalke A, Schulten K (1996) VMD: visual molecular dynamics. J Mol Graph 14: 33-38, 27-38.

11. Friesner RA, Banks JL, Murphy RB, Halgren TA, Klicic JJ, et al. (2004) Glide: a new approach for rapid, accurate docking and scoring. 1. Method and assessment of docking accuracy. J Med Chem 47: 1739-1749.

12. Halgren TA, Murphy RB, Friesner RA, Beard HS, Frye LL, et al. (2004) Glide: a new approach for rapid, accurate docking and scoring. 2. Enrichment factors in database screening. J Med Chem 47: 1750-1759.

13. Sherman W, Day T, Jacobson MP, Friesner RA, Farid R (2006) Novel procedure for modeling ligand/receptor induced fit effects. J Med Chem 49: 534-553.

14. Tina KG, Bhadra R, Srinivasan N (2007) PIC: Protein Interactions Calculator. Nucleic Acids Res 35: W473-476.
